# A novel tumor mutational burden-based risk model predicts prognosis and correlates with immune infiltration in ovarian cancer

**DOI:** 10.3389/fimmu.2022.943389

**Published:** 2022-08-08

**Authors:** Haoyu Wang, Jingchun Liu, Jiang Yang, Zhi Wang, Zihui Zhang, Jiaxin Peng, Ying Wang, Li Hong

**Affiliations:** Department of Obstetrics and Gynecology, Renmin Hospital of Wuhan University, Wuhan, China

**Keywords:** weighted gene correlation network analysis, risk model, ovarian cancer, gene signature, tumor mutational burden (TMB), immune infiltration, immunotherapy

## Abstract

Tumor mutational burden (TMB) has been reported to determine the response to immunotherapy, thus affecting the patient’s prognosis in many cancers. However, it is unclear whether TMB or TMB-related signature could be used as prognostic indicators for ovarian cancer (OC), as its potential association with immune infiltration remains poorly understood. Therefore, this study aimed to develop a novel TMB-related risk model (TMBrisk) to predict the prognosis of OC patients on the basis of exploring TMB-related genes, and to explore the potential association between TMB/TMBrisk and immune infiltration. The mutational landscape, TMB scores, and correlations between TMB and clinical characteristics and immune infiltration were investigated in The Cancer Genome Atlas (TCGA)-OV cohort. Differentially expressed gene (DEG) analyses and weighted gene co-expression network analysis (WGCNA) were performed to derive TMB-related genes. TMBrisk was constructed by Cox regression and further validated in Gene Expression Omnibus (GEO) datasets. The mRNA and protein expression levels and biological functions of TMBrisk hub genes were verified through Gene Expression Profiling Interactive Analysis (GEPIA), GSCA Lite, the Human Protein Atlas (HPA) database, and RT-qPCR. TMBrisk-related biological phenotypes were analyzed in function enrichment and tumor immune infiltration signature. Potential therapeutic regimens were inferred utilizing the Genomics of Drug Sensitivity in Cancer (GDSC) database and connectivity map (CMap). According to our results, higher TMB was associated with better survival and higher CD8+ T cell, regulatory T cell, and NK cell infiltration. TMBrisk was developed based on CBWD1, ST7L, RFX5-AS1, C3orf38, LRFN1, LEMD1, and HMGB1. High TMBrisk was identified as a poor factor for prognosis in TCGA and GEO datasets; the high-TMBrisk group comprised more higher-grade (G2 and G3) and advanced clinical stage (stage III/IV) tumors. Meanwhile, higher TMBrisk was associated with an immunosuppressive phenotype, with less infiltration of a majority of immunocytes and less expression of several genes of the human leukocyte antigen (HLA) family. Moreover, a nomogram containing TMBrisk showed a strong predictive ability demonstrated by time-dependent ROC analysis. Overall, this novel TMB-related risk model (TMBrisk) could predict prognosis, evaluate immune infiltration, and discover new therapeutic regimens in OC, which is very promising in clinical promotion.

## Introduction

Ovarian cancer (OC) is the most lethal gynecological malignancy, with 5-year survival rates below 45%. According to the World Health Organization, it is estimated that the global incidence of OC will be 225,500 cases and 140,200 patients will succumb to this disease each year, making it the seventh most common cancer and the eighth most predominant cause of cancer-related death among women (1, 2). Owing to its insidious outset, rapid development and lack of obvious symptoms, most OC patients are detected at an advanced stage (3). Although the level of diagnosis and treatment has continuously improved, OC remains a serious threat to women’s lives and a salient public concern (4). At present, the International Federation of Gynecology and Obstetrics (FIGO) stage system (3) and some common serum biomarkers, such as carbohydrate antigen 125 (CA125) (5), human epididymis protein 4 (HE4) (6), and breast cancer gene1 (BRAC1) (7), are currently used as diagnostic tools for OC. However, these markers are not proven ideal to evaluate the prognosis and curative effect of each patient precisely (8). There is an obvious need to develop novel and reliable predictive tools for accurate individual evaluation, as well as preselection of suitable treatments.

Human tumors harbor a different number of somatic mutations collectively known as tumor mutational burden (TMB) (9), which is defined as the total number of somatic coding mutations, base substitutions, and insertion–deletion errors per million bases. In recent years, emerging evidence has suggested that TMB can determine the response to immunotherapy, thus affecting the patient’s prognosis (10, 11). One of the primary explanations is that tumor types with high TMB correspond to increased degree of tumor-specific neoantigen generation and presentation, which impacts the strength of immune response (9, 12). Fortunately, OC has been considered one of the most “immunogenic tumors” (13). Immunotherapies have attracted substantial attention and shown promising potential in OC therapy (14). For example, immune checkpoint blockade (ICB) therapy, which inhibits negative regulatory immune checkpoints through various immune checkpoint inhibitors (ICIs), has shown great promise for the treatment of OC (15–17). Due to the promising future of immunotherapy in the treatment of OC and the crucial role of TMB in predicting immunotherapy effect, evaluation of TMB status may be an effective way to predict the prognosis and therapeutic benefit for patients, individually.

**Figure 1:**
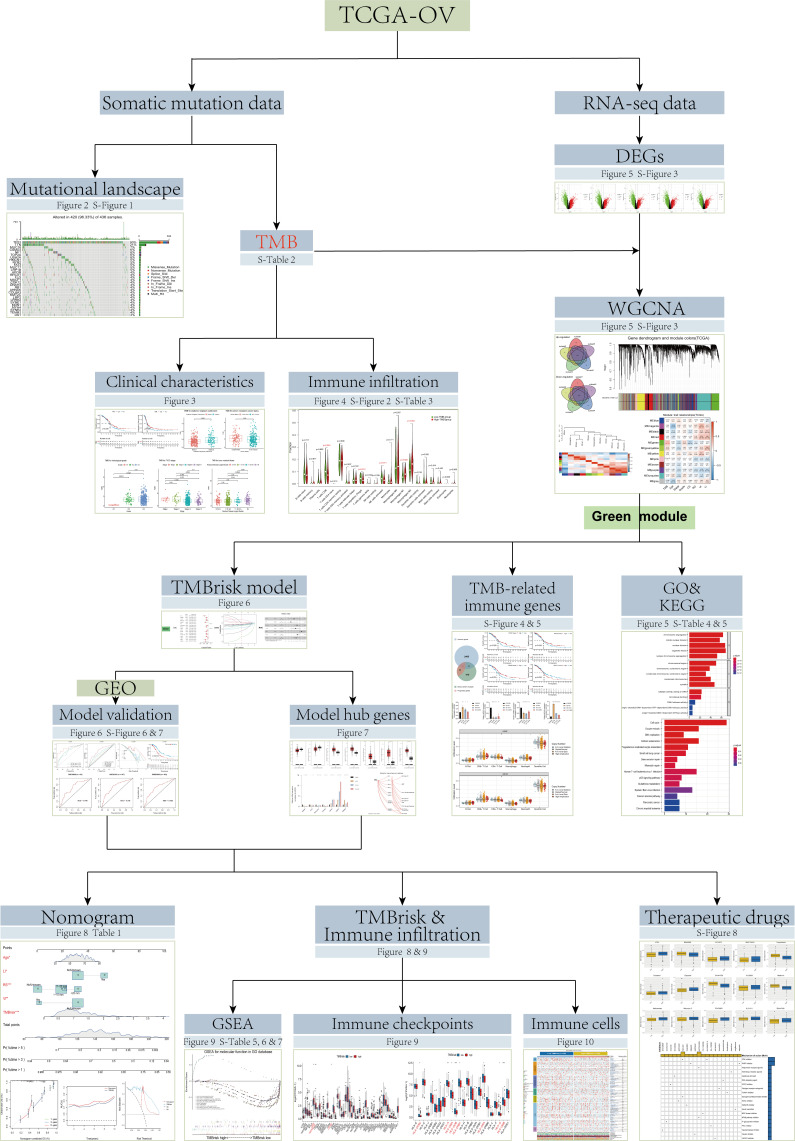
Flowchart of the study.

However, the TMB cutpoints associated with improved curative effect and survival varied markedly between cancer types, and there may not be one universal definition of low/high TMB (11). In addition, determination of TMB is related to whole exome sequencing (WES) of the selected target panel, which increases the difficulty of TMB detection. Coordination and calibration are also required to achieve optimal utility and alignment of all platforms currently in international use (18). Although some studies have established prognostic models based on TMB, the process of screening TMB-related genes can be further optimized (19, 20). Therefore, in the current study, based on the data from The Cancer Genome Atlas (TCGA), Gene Expression Omnibus (GEO), and Genotype-Tissue Expression (GTEx) databases, we screened TMB-related genes by Weighted gene co-expression network analysis (WGCNA) and developed a simplified and practical TMB-related risk model (TMBrisk) to predict survival and immune infiltration in OC patients, which may have higher clinical value. **Figure 1** presents an overview of approach in this study.

## Materials and methods

### Data acquisition and processing

We collected transcriptome profiles (Workflow type: HTseq-Counts), somatic mutation data (Data type: Masked Somatic Mutation; Workflow type:VarScan2) (21), and clinical data of OC patients from Genomic Data Commons Data Portal (https://portal.gdc.cancer.gov/). Due to the absence of matched normal samples in the TCGA database, gene expression data for normal ovarian tissues were downloaded from GTEx portal (http://www.gtexportal.org/home/). For transcriptome profiles, Entrez ID was transformed to the corresponding official gene name with Perl language, and genes with zero expression in more than 50% samples were removed. For mutation analysis, the somatic mutation data in Mutation Annotation Format (MAF) was analyzed and visualized using the “matfools” R package, which offered a number of analysis and visualization modules commonly used in cancer genomic research (22). For clinical data, corresponding information was extracted, including age, histological grade (G1, G2, and G3), FIGO stage (stage I–IV), cancer status (tumor free/with tumor), residual disease largest nodule (no macroscopic disease, 1–10 mm, 11–20 mm, and >20 mm), anatomic neoplasm subdivision (unilateral/bilateral), venous invasion (yes/no), lymphatic invasion (yes/no), Karnofsky performance score, and survival data. Moreover, we selected three validation datasets (GSE18520, GSE26193, and GSE63885) from the GEO database (http://www.ncbi.nlm.nih.gov/geo/) and obtained their normalized microarray gene expression data and clinical data.

### TMB calculation, prognostic analysis, clinical correlation analysis, and immune and stromal scores analysis

In our study, the somatic mutation information was extracted with a Perl script (23), and the TMB score of each sample was calculated through dividing the number of variants by exon length (38 million). For prognostic analysis and clinical correlation analysis, R was utilized to merge the patients’ TMB scores with corresponding clinical information. Kaplan–Meier (K-M) analysis was conducted to compare the difference in overall survival (OS), progression-free survival (PFS), and cancer-specific survival (CSS) with the log-rank test for statistical significance. Wilcoxon rank-sum test was employed to compare clinical traits between two groups, while Kruskal–Wallis test was used among multiple groups. Immune scores, stromal scores, and tumor purity were calculated using the ESTIMATE algorithm (24), which was provided in the “estimate” R package. The correlation between TMB and immune/stromal scores was evaluated using the Pearson method.

### Analysis of immune infiltration for TMB

CIBERSORT, a deconvolution algorithm, can quantify the abundance of any of 22 types of immune cells based on bulk transcriptome profiles (25). We normalized the transcriptome data of OC patients with the “limma” R package (26) and uploaded the prepared data to the CIBERSORT algorithm (R script v1.03) to evaluate the distribution of different immune cells in each sample. The Tumor Immune Estimation Resource (TIMER) database (http://timer.comp-genomics.org/) (27) includes 10,897 samples from 32 cancer types in TCGA and pre-calculates the infiltration levels of six types of immune cells. In the current study, to explore the prognostic value of immune cells, we used the “Survival” module to output tumor-infiltrating immune cell (TIIC)-related K-M plots (27). Furthermore, the “Somatic Copy Number Alterations (SCNA)” module was used to assess the relationship between different SCNAs of genes and immune infiltration. The SCNAs were defined by GISTIC 2.0, including deep deletion, arm-level deletion, diploid/normal, arm-level gain, and high amplification (28).

### Differentially expressed gene screening

We utilized the “limma” R package (26) to identify DEGs between OC and normal samples with the criteria of fold change (FC) > 2 and false discovery rate (FDR) < 0.05. Since the tumor sample size (n = 379) was much larger than that of the normal sample (n = 80), we implemented a subset-based strategy to balance the sample size in order to make the screening result more objective and accurate (29). In detail, five random samplings were performed. For each random sampling, a subset of 100 patients were selected from the tumor group without repetition. DEGs were identified by analyzing differences in transcriptome profiles between each tumor subset (n = 100) and normal samples (n = 80). The intersection of DEGs obtained from five independent analyses was selected as the final DEGs between the two groups. Volcano plots of five analyses were drawn by the “ggplot2” R package. Venn diagrams were plotted by the “VennDiagram” R package to exhibit the common DEGs.

### Weighted gene co-expression network construction

In the current study, a scale-free gene co-expression network was constructed using the “WGCNA” in R software to recognize co-expressed gene modules closely related to TMB (30). First, to ensure the reliability of network construction, sample clustering was carried out to distinguish and remove outlier samples. By calculating the corresponding scale independence (R²) and mean connectivity when the soft threshold ranged from 1 to 20, the optimal value of soft threshold was determined. Based on Pearson’s coefficients of pairwise gene correlations and the chosen soft threshold value, a weighted adjacency matrix was constructed and subsequently transformed into a topological overlap matrix (TOM) (31). Next, the corresponding dissimilarity matrix (1-TOM) was used to establish a hierarchically gene clustering tree. Genes in the same branch of the dendrogram were highly correlated and clustered into the same co-expression module using the dynamic tree cut method, with a minimum module size cutoff of 50 and a deepSplit value of 2. Highly similar modules were screened and merged together with the height cutoff of 0.25. In addition, the relationship between each module and clinical parameters was analyzed by Pearson correlation analysis and visualized by a heatmap. Gene modules of |correlation coefficient| > 0.2 and p value < 0.05 were considered as strong TMB-correlated modules.

### Functional enrichment analysis for co-expressed gene modules

To explore the biological functions and pathways of modules, we obtained the Entrez ID of each gene via the “org.Hs.eg.db” R package and conducted Gene Ontology (GO) and Kyoto Encyclopedia of Genes and Genomes (KEGG) analyses using the “clusterProfiler,” “enrichplot,” “ggplot2”, and “Goplot” R packages (32).

### Identification of TMB-related immune genes

We downloaded a list of 2,483 immune-related genes from the Immunology Database and Analysis Portal (Immport) (https://www.immport.org/shared/genelists/) (33, 34), and selected genes that overlapped with DEGs in the green module as TMB-related immune genes. The result was visualized using the “VennDiagram” R package. Then, batch survival analysis was performed via a “for cycle” R script to screen candidate genes associated with survival outcomes for further investigation.

### Development of TMB-related risk model

As a preprocessing step, expression values of genes were log2 transformed after adding a pseudo-count of 1. Univariate Cox regression analyses, least absolute shrinkage and selector operation (LASSO) regression analyses, multivariate Cox regression analyses, and Akaike information criterion (AIC)-based stepwise Cox regression analyses were successively used to screen prognostic genes for constructing the risk model. LASSO is a regularization and descending dimension method that can be used in conjunction with Cox models for biomarker screening (35). The model with minimal AIC value was determined as the final model, established by multiplying mRNA levels of each gene by respective multivariate Cox regression coefficient.

### Validation of expression patterns and identification of signaling pathways of hub genes

The mRNA expression patterns of the TMBrisk hub genes were verified by Gene Expression Profiling Interactive Analysis (GEPIA) (http://gepia.cancer-pku.cn/) (36). The protein expression of the hub genes between OC and normal tissues was determined using immunohistochemistry (IHC) from the Human Protein Atlas (HPA) (https://www.proteinatlas.org/), which is a valuable database providing extensive transcriptome and proteomic data for specific human tissues and cells. GSCA Lite database (http://bioinfo.life.hust.edu.cn/web/GSCA Lite/) (37), a web-based platform for Gene Set Cancer Analysis, was employed for statistics of deletion/amplification of hetero/homozygous copy number variation (CNV) and identification of OC-related signaling pathways of hub genes. Due to the lack of RFX5-AS1 in GEPIA, HPA, and GSCA Lite, RFX5 was used as a substitute for analysis.

### Development of the nomogram

We performed the nomogram with the independent prognostic factors screened by multivariate Cox analysis via the “rms” R package. To evaluate the prediction accuracy of the nomogram, the “survival” R package was used to calculate Harrell’s Concordance index (C-index) to quantify its discrimination performance. The calibration curves of survival probability for different years were plotted using the Hosmer–Lemeshow test.

### Functional enrichment analysis for TMBrisk

In order to explore TMBrisk-related pathways without the restriction of DEGs, gene set enrichment analysis (GSEA) was implemented with GSEA software 4.2.2 (https://www.gsea-msigdb.org/gsea/index.jsp) (38). We used TMBrisk as the phenotype; “c5.go.mf.v7.5.1.symbols.gmt”, “c5.go.bp.v7.5.1.symbols.gmt”, and “c2.cp.kegg.v7.5.1.symbols.gmt” as the reference gene set, which were obtained from the Molecular Signatures Database (MSigDB) (http://software.broadinstitute.org/gsea/msigdb/) (39). The significant pathways were defined as those whose |normalized enriched score (NES)| > 1 and FDR < 0.05.

### Analysis of tumor immune signatures for TMBrisk

On the one hand, we evaluated the expression of the human leukocyte antigen (HLA) gene family and the immune checkpoints (40, 41). On the other hand, the levels of infiltrating immune cells and stromal cells were calculated by seven algorithms ]TIMER (27), CIBERSORT (25), xCell (42), CIBERSORT-ABS (43), QUANTISEQ (44), MCPCOUNTER (45), and EPIC (46)]. The results are available on the TIMER database.

### Prediction of treatment sensitivity and small molecule drugs

Chemotherapeutic response in OC patients was assessed utilizing the Genomics of Drug Sensitivity in Cancer (GDSC) database (https://www.cancerrxgene.org) (47). The 50% inhibiting concentration (IC50) value of the 138 drugs in GDSC was inferred using the pRRophetic algorithm. Possible small-molecule drugs for OC were forecasted using the Connectivity map (CMap) (https://www.broadinstitute.org/connectivity-map-cmap) (48), which was premised on DEGs between low- and high-TMBrisk groups with |FC| > 2 and FDR < 0.05.

### Cell lines and cell culture

OC cells A2780, SKOV3, and OVCAR3, and normal ovarian surface epithelium cells IOSE80 were purchased from the Cell Storage Center of Wuhan University (Wuhan, China). A2780, SKOV3, and OVCAR3 cells belong to the human epithelial OC cell line, and IOSE80 cells as normal control were also from ovarian surface epithelium. A2780, IOSE80, and OVCAR3 were grown in RPMI-1640 medium (Hyclone) supplemented with 10%, 10%, and 20% fetal bovine serum, respectively. SKOV3 were grown in McCoy’s 5A medium (Biological Industries) supplemented with 10% fetal bovine serum. Cells were incubated with 5% CO2 atmosphere at 37°C.

### Quantification of gene expression by real-time quantitative PCR

Total mRNA was purified using TRIzol reagent (TaKaRa) according to the manufacturer’s instructions. To generate cDNA, 2 μg of RNA was subjected to reverse transcription using the Hifair®II1st Strand cDNA Synthesis SuperMix (YEASEN). Real-time quantitative polymerase chain reaction (RT-qPCR) was performed with Hieff® qPCR SYBR Green Master Mix (YEASEN), using a CFX Connect Real-Time Cycler (Bio-Rad). GAPDH was set as an internal control for gene quantification. Technical and biological replicates of each gene were performed at least three times during RT-qPCR analysis. **Supplementary Table 1** contains the RNA molecules evaluated on cell lines and their corresponding primers.

### Survival and other statistical analysis

K-M survival analyses were tested by log rank. Receiver operating characteristic (ROC) curves were plotted by the “survivalROC” R package to evaluate the accuracy of TMBrisk and nomogram. Decision curve analysis (DCA) was conducted using “ggDCA” R package to assess the clinical outcomes of different decision strategies (49). All statistical analyses were completed by R software (version 3.6.3). R packages used in our study are mentioned above. Benjamini–Hochberg for multiple testing and false discovery rate (FDR) were used to correct the p-value. The Spearman method was used to calculate the correlation coefficient and p-value in the correlation analysis of TMBrisk and other variables. Wilcoxon rank-sum test and Kruskal–Wallis test were used for subgroup differential analyses. Two-tailed tests and p values < 0.05 for significance were used.

## Results

### Landscape of mutation profiles in OC samples

**Figure 2:**
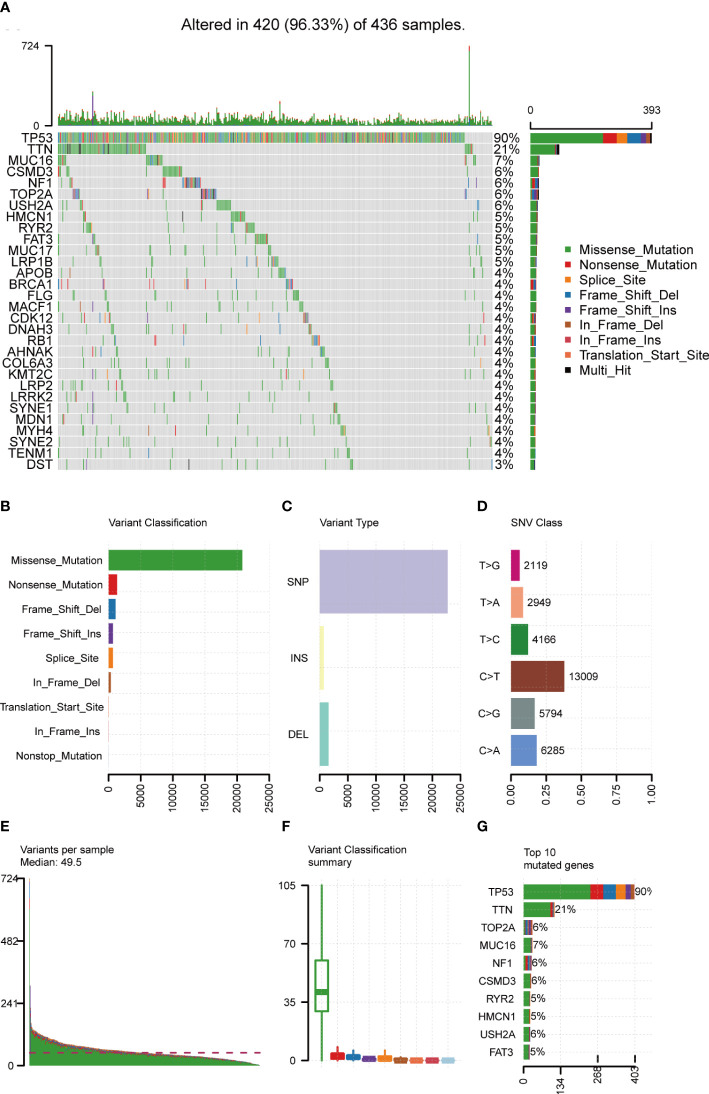
Landscape of mutation profiles in OC samples. **(A)** Mutation information of each gene in each sample was shown in the waterfall plot, with various color annotations to distinguish different mutation types. The barplot above the legend exhibited the mutation burden, and the other barplot on the right showed the distribution of mutation types among the top 30 genes. **(B–D)** According to different classification categories, missense mutation, SNP, and C > T mutation accounted for a larger proportion. **(E)** Mutation burden in each sample. **(F)** The summary of the occurrence of each variant classification. **(G)** Top 10 mutated genes in ovarian cancer. SNP, single-nucleotide polymorphism; SNV, single-nucleotide variant.

Mutation data of 436 OC patients were downloaded from the TCGA database and the “maftools” R package was implemented to analyze and visualize the landscape of mutation profiles. The different genetic mutations in each sample were shown in the waterfall plot, with various color annotations representing different mutation types (**Figure 2A**). The mutations were further classified into different categories. By comparison, missense mutations accounted for the largest fraction (**Figure 2B**), single-nucleotide polymorphisms (SNPs) were more frequent than insertions or deletions (**Figure 2C**), and C>T represented the most common type of single-nucleotide variant (SNV) (**Figure 2D**). Moreover, counting the number of altered bases per patient, we found that the median and maximum number of mutations were 49.5 and 724, respectively (**Figure 2E**). For each variant classification, the number of occurrences is shown in the box plot (**Figure 2F**). Furthermore, taking into consideration the total number of mutations and counting the multiple hits alone, we recalculated the top 10 mutated genes (**Figure 2G**), which were slightly different from the previous ones (**Figure 2A**). Finally, the co-occurrence and exclusive associations across mutated genes were investigated. Significant co-occurrences were observed among mutations of COL6A3 and DNAH3, APOB and MUC16, etc. (**Supplementary Figure 1**).

### Calculation of TMB and its correlation with prognosis and clinical traits

**Figure 3:**
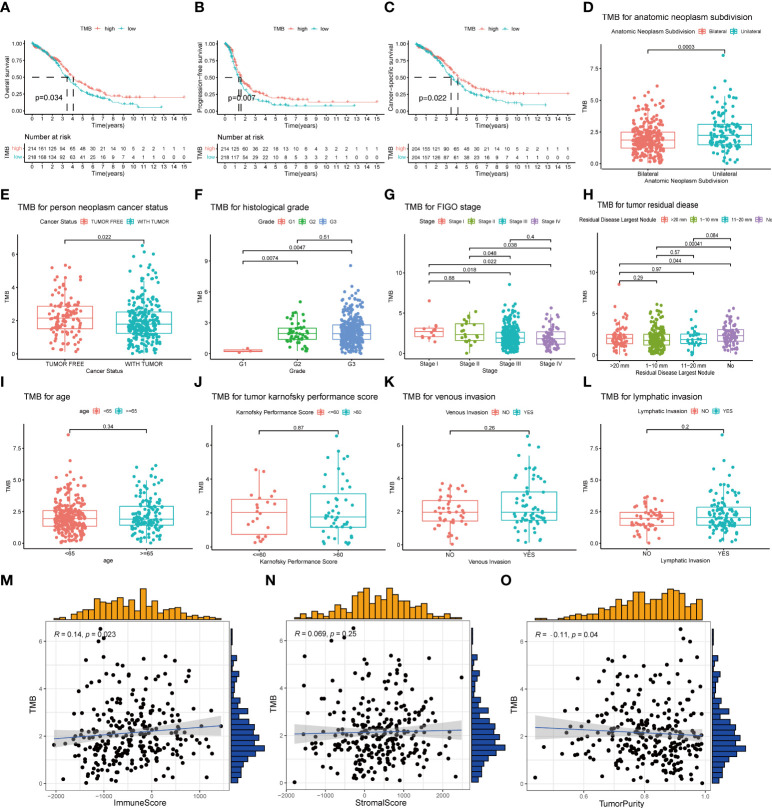
Association of TMB with prognosis and clinical traits. **(A–C)** Lower TMB indicated a better OS, PFS, and CSS with p = 0.034, p = 0.007, and p = 0.022, respectively**. (D, E)** Unilateral subdivision and tumor-free status correlated with higher TMB level. **(F–H)** Higher TMB level was associated with higher histological grades, lower FIGO stages, and smaller residual tumor sizes. **(I–L)** No significant differences were observed with age, Karnofsky performance score, or venous/lymphatic invasion. **(M–O)** TMB significantly correlated with immune scores and tumor purity but not with stromal scores. TMB, tumor mutational burden; OS, overall survival; PFS, progression-free survival; CSS, cancer-specific survival; FIGO, International Federation of Gynecology and Obstetrics.

A total of 436 OC patients in the TCGA database were included in our study to calculate TMB scores. The range of TMB spanned from 0.026 to 29.289 (**Supplementary Table 2**). For an improved accuracy of the analysis, one statistical outlier (TMB = 29.289) was removed. To assess the relationship between TMB and prognosis, patients were divided into low- and high-TMB groups with the median score as the cutoff value. According to the K-M analysis, the high-TMB group showed a trend toward better OS, PFS, and CSS than the low-TMB group (**Figures 3A–C**). For clinical characteristics, TMB had a significant distinct distribution in the patients presented with bilateral/unilateral tumors (**Figure 3D**) and patients with tumor or tumor-free patients (**Figure 3E**). Significant correlations also existed between TMB and histological grades (G1 vs. G2, p < 0.01; G1 vs. G3, p < 0.01) (**Figure 3F**), FIGO stages (SI vs. SIII, p = 0.018; SI vs. SIV, p = 0.022; SII vs. SIII, p = 0.048; SII; vs. SIV, p = 0.038) (**Figure 3G**), and residual disease (NO vs. 1–10 mm, p < 0.01; NO vs. >20 mm, p = 0.044) (**Figure 3H**), which indicated that higher TMB was associated with higher histological grades, lower clinical stages, and smaller residual tumor sizes. However, no significant association was found between TMB and age, Karnofsky performance score, or venous/lymphatic invasion (**Figures 3I–L**). Furthermore, TMB significantly correlated with immune scores and tumor purity inferred by ESTIMATE algorithms with positive and negative dependencies, respectively (**Figures 3M, N**), but there was no significant association with stromal scores (**Figure 3O**).

### Evaluation of immune infiltration in the low- and high-TMB groups

**Figure 4:**
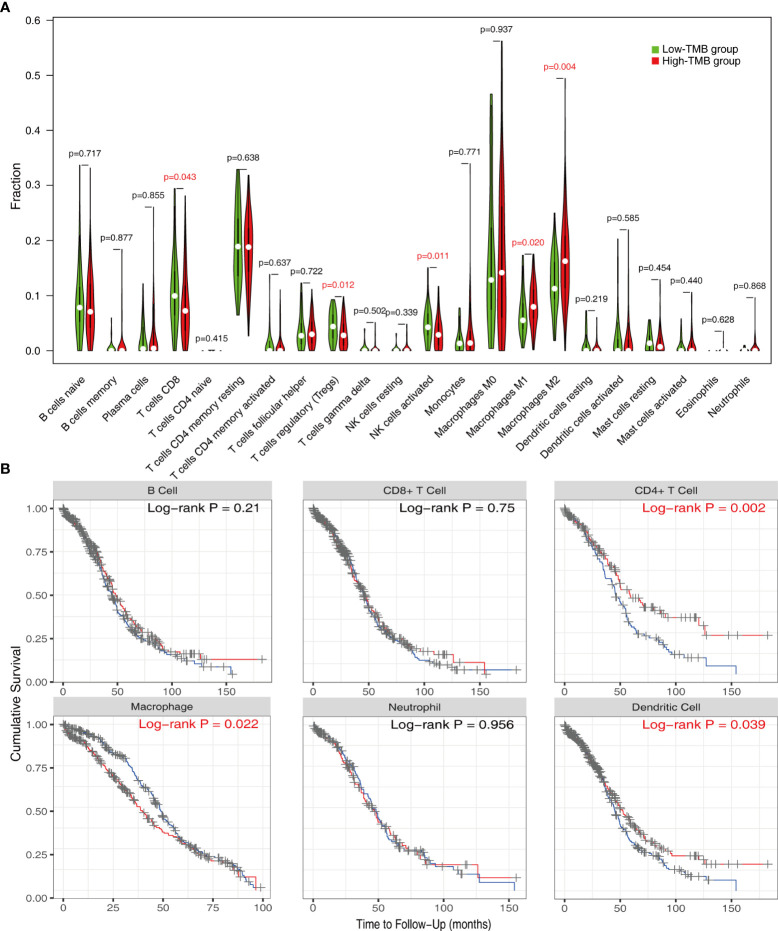
Evaluation of immune infiltration in the low- and high-TMB groups. **(A)** Comparison of abundance of 22 infiltrating immune cells between low- and high-TMB groups. **(B)** K-M analysis revealed that high infiltration levels of CD4+ T cells and dendritic cells and low infiltration levels of macrophage were associated with better survival outcomes. TMB, tumor mutational burden.

In order to better understand the potential effects of TMB on immune activity and response, we compared immune infiltration between the low- and high-TMB groups. Based on the CIBERSORT algorithm, we estimated the proportion of 22 types of immune cells in each sample (**Supplementary Table 3**). After the filtration of samples with p < 0.05, the distribution of infiltrating immune cells in 192 samples was shown in a barplot (**Supplementary Figure 2**). Then, we analyzed the difference in the proportion of each type of immune cell between two TMB groups. Wilcoxon rank-sum test revealed that compared with the low-TMB group, CD8+ T cells (p = 0.043), regulatory T cells (Tregs) (p = 0.012), and activated natural killer (NK) cells (p = 0.011) exhibited lower infiltrating levels in the high-TMB group. However, samples in the high-TMB group had a significant increase in the fraction of macrophages M1 (p = 0.02) and macrophages M2 (p = 0.004) (**Figure 4A**).

Reportedly, immune infiltration could affect the prognosis of patients. Therefore, we performed K-M analysis on the six types of immune cells and found that high infiltration levels of CD4+ T cell and dendritic cell were associated with a favorable prognosis, while the situation was reversed in macrophage (**Figure 4B**).

### Construction of co-expression modules by WGCNA

**Figure 5:**
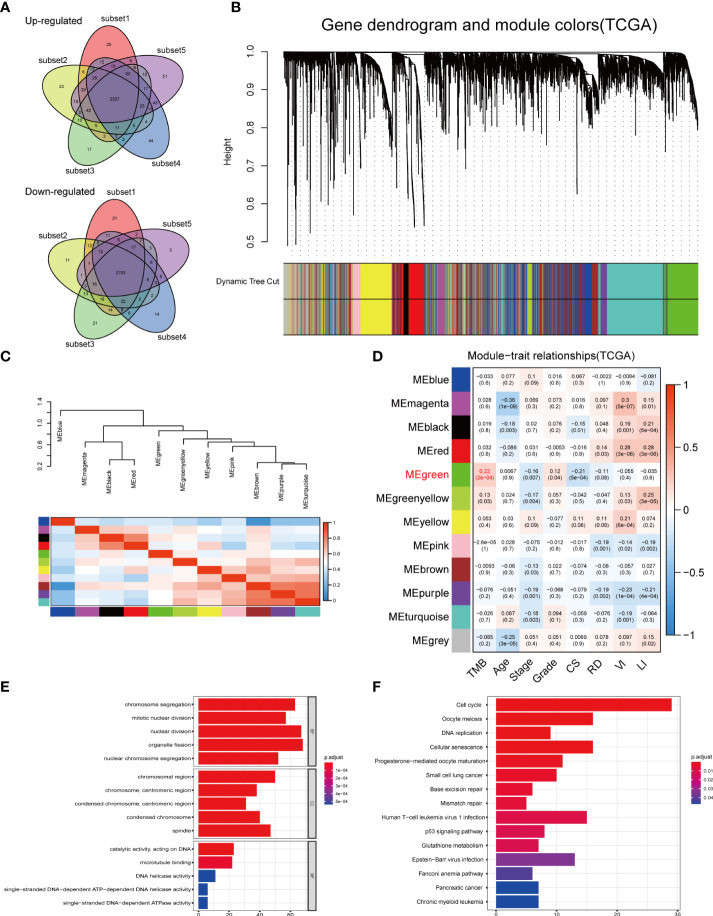
DEGs analysis and construction of co-expression modules. **(A)** Venn diagrams showing the common upregulated genes and common downregulated genes from five subset-based analyses. **(B)** Clustering dendrograms of DEGs using the dissimilarity measure (1-TOM), with assigned module colors. **(C)** Visualizing MEs with the hierarchical clustering plot. **(D)** Correlation between gene modules and clinical traits. Each row corresponded to a module eigengene, and each column to a trait. **(E)** GO and **(F)** KEGG enrichment analysis of the green module. DEGs, differentially expressed genes; TOM, topological overlap matrix; MEs, module eigengenes; CS, cancer status; RD, residual disease; VI, venous invasion; LI, lymphatic invasion; GO, Gene Ontology; BP, biological process; CC, cellular component; MF, molecular function; KEGG, Kyoto Encyclopedia of Genes and Genomes.

The high gene mutation rate in OC prompted us to explore the key genes that significantly influenced the overall mutational load. Through a subset-based method, differential gene expression analyses were repeated five times (**Supplementary Figure 3A**). The Venn diagrams showed the intersection of DEGs obtained from five analyses (**Figure 5A**). Between the normal and OC samples, a total of 5,120 DEGs were identified, including 2,337 upregulated genes and 2,783 downregulated genes. These DEGs were used for WGCNA. In WGCNA, no outlier samples were removed according to the sample clustering result, and the trait heatmap showed the distribution of samples according to their corresponding clinical characteristics (**Supplementary Figure 3B**). The optimal soft threshold value of 5 was chosen to guarantee the scale-free distribution (**Supplementary Figure 3C**). According to WGCNA results, 11 co-expression modules were recognized (except the gray module containing genes that were not co-expressed) (**Figure 5B**), and the hierarchical clustering plot revealed module eigengenes (MEs) (**Figure 5C).** The green module had the strongest correlation with TMB (|Cor| > 0.2, p < 0.001) (**Figure 5D**), which was also demonstrated by the scatter plot showing a significant correlation between module membership (MM) and gene significance (GS) (**Supplementary Figure 3D**).

GO and KEGG enrichment analyses were conducted to uncover the potential biological functions of genes in the green module. According to GO enrichment results, the genes played important roles in cell proliferation-related pathways, such as organelle fission, nuclear division, and chromosome segregation (**Figure 5E**, **Supplementary Table 4**). KEGG analysis revealed 15 significant signaling pathways, including cell cycle, oocyte meiosis, and cellular senescence (**Figure 5F**, **Supplementary Table 5**). These results implied that mutations in these genes may be potentially critical for tumorigenesis.

Furthermore, we sought to gain a deeper insight into TMB-related immune genes. Four immune genes (HDGF, NRAS, PSMD6, and NR1D1), which were significantly correlated with survival, were identified between the intersection of the immune gene set obtained from Immport and the green module (**Supplementary Figure 4A**). K-M curves showed that the high expression level of HDGF, NRAS, and PSMD6 was associated with an improved prognosis, while the opposite happened for NR1D1 (**Supplementary Figure 4B**). RT-qPCR indicated that HDGF and NRAS were overexpressed in OC, while NR1D1 and PSMD6 were underexpressed (**Supplementary Figure 4C).** We also used the HPA database to explore the differences in protein levels of the four immune genes between normal and tumor tissues (**Supplementary Figure 4D**). More importantly, the impact of copy number alteration of the four genes on immune infiltration in OC patients was evaluated. In brief, compared to patients with diploid/normal expression of HDGF, NR1D1, and NRAS, patients with high copy number amplification of HDGF or copy number deletion of NR1D1/NRAS had a lower level of immune infiltration, including CD8+ T cells, CD4+ T cells, and neutrophils. On the other hand, decreased CNV of PSMD6 upregulated immune infiltration, especially of macrophages (**Supplementary Figure 5**).

### Establishment and validation of the TMBrisk model

**Figure 6:**
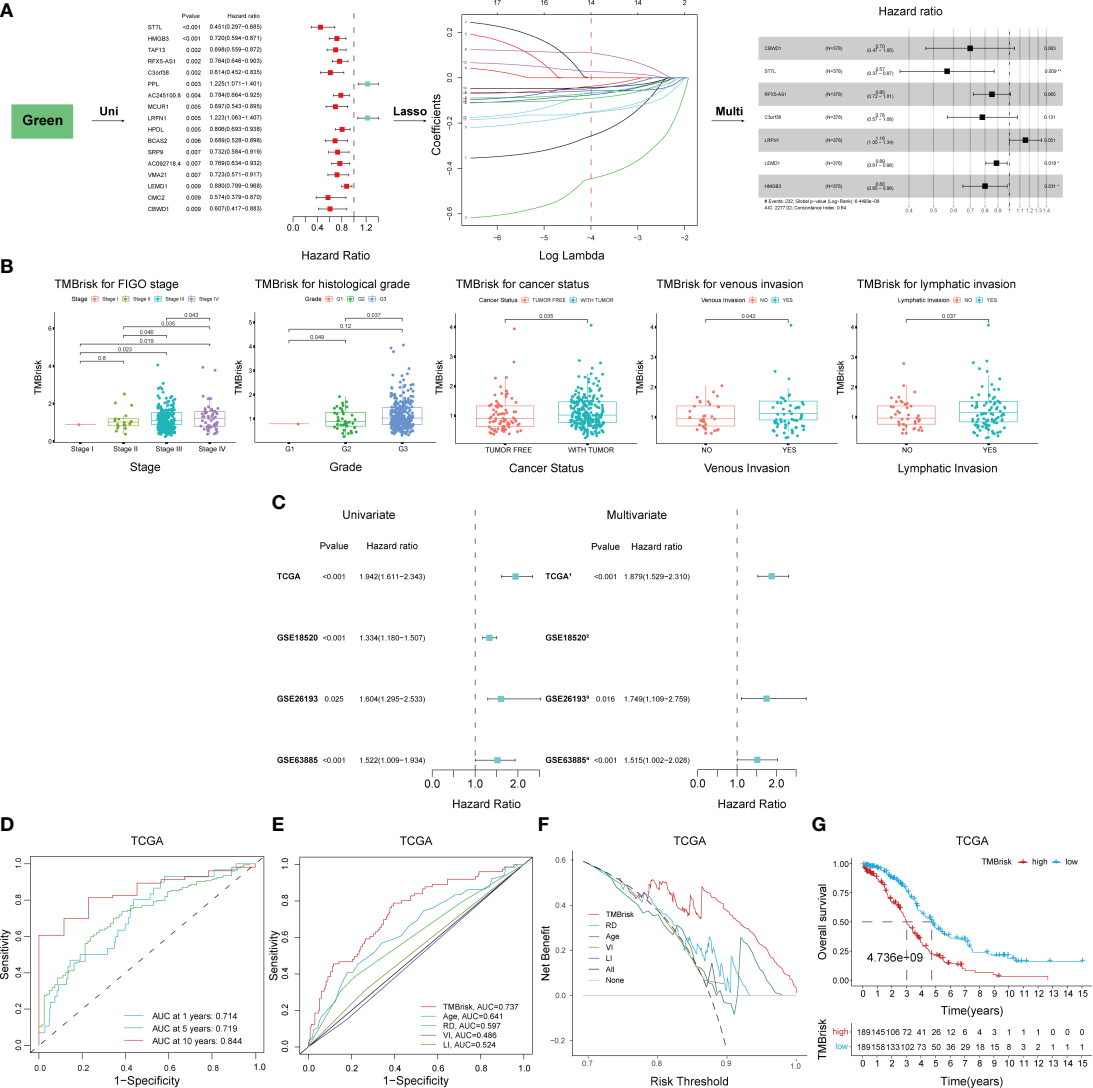
Establishment and validation of the TMBrisk model. **(A)** Cox regression analyses for selecting gene signatures. The first forest plot showing genes with p < 0.01 in univariate Cox analysis. These genes were further inputted into LASSO analysis. The y-axis showed LASSO coefficients and the x-axis was –log (lambda). Dotted vertical lines represented 1 standard error values of lambda. The genes selected at 1 standard error values of lambda were finally used for multivariate Cox analysis, with the second forest plot showing the best signature. **(B)** Difference analysis of the distribution of TMBrisk in different clinical characteristics. **(C)** Forest plot of Cox analysis in TCGA and GEO datasets. (1) For multivariate Cox regression analysis, HR value of TMBrisk was adjusted by age, FIGO stage, histological grade, residual disease, cancer status, venous invasion, and lymphatic invasion. (2) The multivariate analysis was not performed in GSE18520 because of missing clinical information. (3) For multivariate Cox regression analysis, HR value of TMBrisk was adjusted by FIGO stage and histological grade. (4) For multivariate Cox regression analysis, HR value of TMBrisk was adjusted by residual tumor size, FIGO stage, and histological grade. **(D)** ROC curves of TMBrisk for 1-, 5-, and 10-year survival prediction in TCGA. **(E)** ROC curves of TMBrisk and other prognostic predictors for 2-year survival prediction in TCGA. **(F)** DCA for TMBrisk and other prognostic predictors. The y-axis represented net benefit and the x-axis represented risk threshold. The black line represented the hypothesis that all patients had OC. The gray line represented the hypothesis that no patient had OC. **(G)** High-TMBrisk group correlated with poor survival outcome in TCGA, with p < 0.0001. TMB, tumor mutational burden; LASSO, Least Absolute Shrinkage and Selection Operator; FIGO, International Federation of Gynecology and Obstetrics; ROC, receiver operating characteristic curve; RD, residual disease; VI, venous invasion; LI, lymphatic invasion; DCA, decision curve analysis; HR, hazard ratio; 95% CI, 95% confidence interval.

Given the significant correlation between TMB and prognosis and the limitations of TMB, we considered to establish a novel risk model based on TMB-related DEGs. The 496 genes in the green module were first inputted into univariate Cox regression analysis, which identified 17 genes with p < 0.01 (**Figure 6A**); then, they were further screened by LASSO Cox regression (**Figure 6A**). The remaining 14 genes were finally enrolled in multivariate Cox analysis with stepwise regression. The model consisting of CBWD1, ST7L, RFX5-AS1, C3orf38, LRFN1, LEMD1, and HMGB3 had a minimal AIC value (AIC = 2,277.02) (**Figure 6A**). Therefore, the TMB-related risk model (TMBrisk) was established as follows: TMBrisk = (−0.3585) * CBWD1 + (−0.5693) * ST7L + (−0.1593) * RFX5-AS1 + (−0.2473) * C3orf38 + (0.1461) * LRFN1 + (−0.1178) * LEMD1 + (−0.2224) * HMGB3.

To elucidate the underlying role of TMBrisk in the development of OC, we investigated the distribution of TMBrisk in patients with different clinical characteristics. We found that tumor-bearing patients and patients with higher histological grades, advanced FIGO stages, venous invasion, or lymphatic invasion had a higher TMBrisk score (**Figure 6B**). Then, univariate and multivariate Cox regression analyses were conducted utilizing TCGA and three GEO datasets (GSE18520, GSE26193, and GSE63885) to examine the significance of the impact of TMBrisk on prognosis. The outcomes illustrated that in the four datasets, a higher TMBrisk score indicated poorer OS, and TMBrisk was identified as an independent prognostic factor after adjusting other clinical factors (**Figure 6C**). ROC curves of OS prediction in the four datasets showed that TMBrisk had a strong predictive value with the area under the curve (AUC) > 0.7 (**Figure 6D**, **Supplementary Figures 6A–C**). For 2-year OS prediction in TCGA, the AUC of TMBrisk was also significantly higher than that of other independent prognostic predictors (**Figure 6E**). Meanwhile, the DCA for 10-year OS prediction showed that TMBrisk had the highest net benefit (**Figure 6F**). Subsequently, patients in TCGA and three validation datasets were divided into low- and high-TMBrisk groups based on the median score, respectively. K-M curves showed that patients in the low-TMBrisk group exhibited improved OS, PFS, and CSS than those in the corresponding high-TMBrisk group (**Figure 6G**, **Supplementary Figures 6D–H**). The result of stratified analysis revealed significant differences in OS between low- and high-TMBrisk for subgroups with different age, FIGO stage, histological grade, residual disease, venous invasion, lymphatic invasion, and anatomic subdivision (**Supplementary Figure 7**). Taken together, the above results indicated that the risk model had excellent predictive ability, robust performance and potential clinical application value.

### Validation of the expression of TMBrisk hub genes

**Figure 7:**
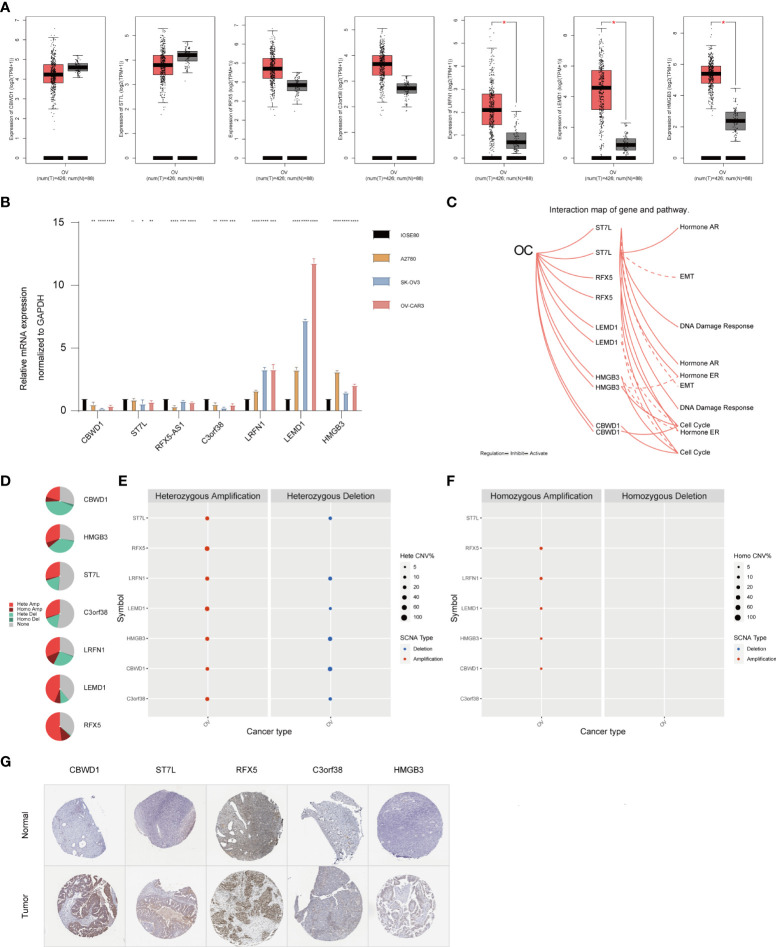
Expression pattern validation and relevant biological functions of hub genes in OC. **(A)** Expression pattern of CBWD1, ST7L, RFX5, C3orf38, LRFN1, LEMD1, and HMGB3 in OC and normal samples from the GEPIA database. **(B)** RT-QPCR analysis of CBWD1, ST7L, RFX5-AS1, C3orf38, LRFN1, LEMD1, and HMGB3. **(C)** Related signaling pathways of ST7L, RFX5, LEMD1, HMGB3, and CBWD1 in OC. (D–F) CNV of CBWD1, ST7L, RFX5, C3orf38, LRFN1, LEMD1, and HMGB3 in OC from the GSCA Lite database, including heterozygous **(E)** and homozygous **(F)** CNV. **(G)** Immunohistochemistry of CBWD1, ST7L, RFX5, C3orf38, and HMGB3 in OC and normal samples from the HPA database. *p < 0.05; **p < 0.01; ***p < 0.001; ****p < 0.0001. CNV, copy number variation.

To verify the reliability of the seven hub genes that made up the TMBrisk model, we validated the expression patterns of these genes through databases and RT-qPCR. According to GEPIA, mRNA expression levels of LRFN1, LEMD1, and HMGB3 were significantly higher in OC than in normal samples. The mRNA expression levels of CBWD1, ST7L, RFX5, and C3orf38 were decreased in OC, although not significantly (**Figure 7A**). RT-qPCR demonstrated that OC cells had higher mRNA expression of LRFN1, LEMD1, and HMGB3, and lower mRNA expression of the other five genes compared with control cells, which was consistent with the results of GEPIA (**Figure 7B**). In addition, according to HPA, the protein expression levels of the five genes (data for LRFN1 and LEMD1 were lacking and therefore not presented) were significantly higher in tumor samples than in normal samples (**Figure 7G**). Finally, we analyzed the signaling pathways and CNV of these genes in GSCA Lite. ST7L was found to be activated and inhibited in DNA damage response pathway and EMT pathway of OC, respectively. In the cell cycle pathway, RFX5, CBWD1, and HMGB3 were activated, while LEMD1 was inhibited. As for hormone-related pathways, ST7L was activated in the hormone AR/ER pathways, and HMGB3 was inhibited in the hormone ER pathway (**Figure 7C**). These pathways played vital roles in oncogenesis, suggesting that the hub genes may participate in OC progression. Furthermore, HMGB3 and LRFN1 had a high proportion of both heterozygous amplification and heterozygous deletion, suggesting that HMGB3 and LRFN1 may be the characteristic genes of OC. RFX5 and LEMD1 were characterized by high heterozygous amplification, while CBWD1 harbored more heterozygous deletion (**Figures 7D–F**).

### Construction and validation of nomogram for survival prediction

**Figure 8:**
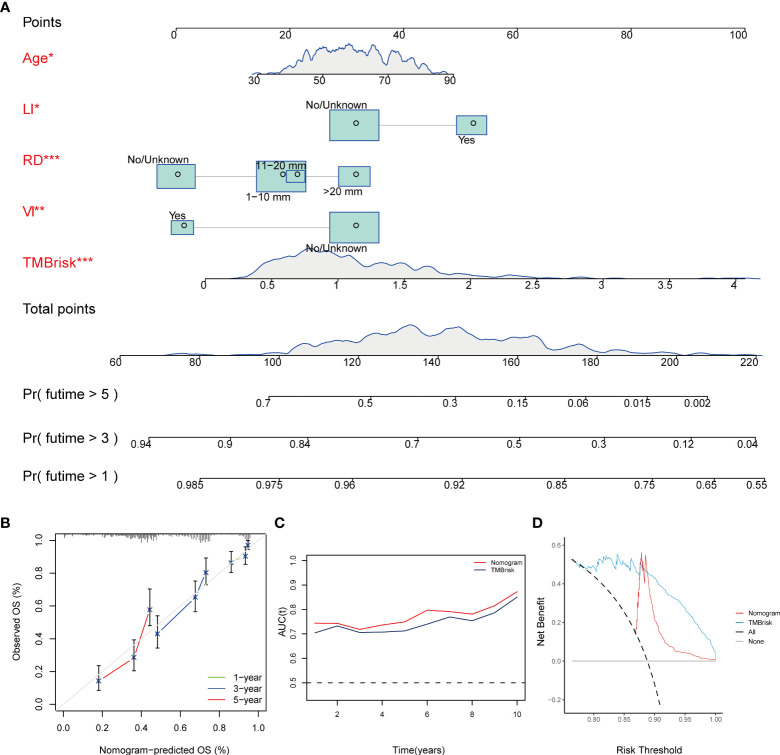
Construction and validation of nomogram. **(A)** Nomogram constructed based on TMBrisk, age, residual disease, venous invasion, and lymphatic invasion as predictive factors to predict 1-, 3-, and 5-year survival probability. **(B)** Calibration curves for the survival probability at 1, 3, and 5 years. **(C)** Time-dependent AUC value of the nomogram and TMBrisk in TCGA. **(D)** DCA curves to evaluate the clinical utility of different decision strategies. *p < 0.05; **p < 0.01; ***p < 0.001. TMB, tumor mutational burden; RD, residual disease; VI, venous invasion; LI, lymphatic invasion; AUC (t), time-dependent AUC; DCA, decision curve analysis.

Multivariate Cox regression analysis performed in the TCGA dataset screened the TMBrisk, age, residual disease, venous invasion, and lymphatic invasion as independent prognostic factors (**Figure 6D**, **Table 1**). To strengthen predictive power, we constructed a nomogram based on the above prognostic factors (**Figure 8A**). The calibration curves for the survival possibility at 1, 3, and 5 years exhibited accurate prediction ability of the nomogram (**Figure 8B**). Time-dependent AUC suggested that in comparison to the TMBrisk, the nomogram further enhanced the prediction ability (**Figure 8C**). DCA showed that the nomogram and TMBrisk provided better net benefit over different threshold probability ranges, respectively, suggesting that both might have potential applications in different contexts (**Figure 8D**). Taken together, the above results indicated that the nomogram had enhanced prediction efficiency, as well as potential clinical application value.

**Table 1:** Univariate and multivariate Cox regression analyses of clinical features and TMBrisk with OS in TCGA.

Variables	Univariate analysis			Multivariate analysis		
	HR	95% CI of HR	p-value	HR	95% CI of HR	p-value
Age	1.022	1.009–1.034	<0.001	1.015	1.002–1.028	0.026
Histological grade	1.217	0.813–1.822	0.340	1.052	0.695–1.593	0.809
FIGO stage	1.237	0.924–1.657	0.154	1.089	0.797–1.487	0.593
Residual disease	1.334	1.180–1.507	<0.0001	1.263	1.108–1.440	<0.001
Venous invasion	0.690	0.455–1.047	0.081	0.463	0.261–0.820	0.008
Lymphatic invasion	1.104	0.795–1.533	0.555	1.749	1.109–2.759	0.016
Anatomic subdivision	1.082	0.811–1.443	0.594	1.118	0.825–1.513	0.472
TMBrisk	1.942	1.611–2.343	<0.0001	1.879	1.528–2.310	<0.0001

HR, hazard ratio; CI, confidence interval.

### Association of TMBrisk with OC immune signature

**Figure 9:**
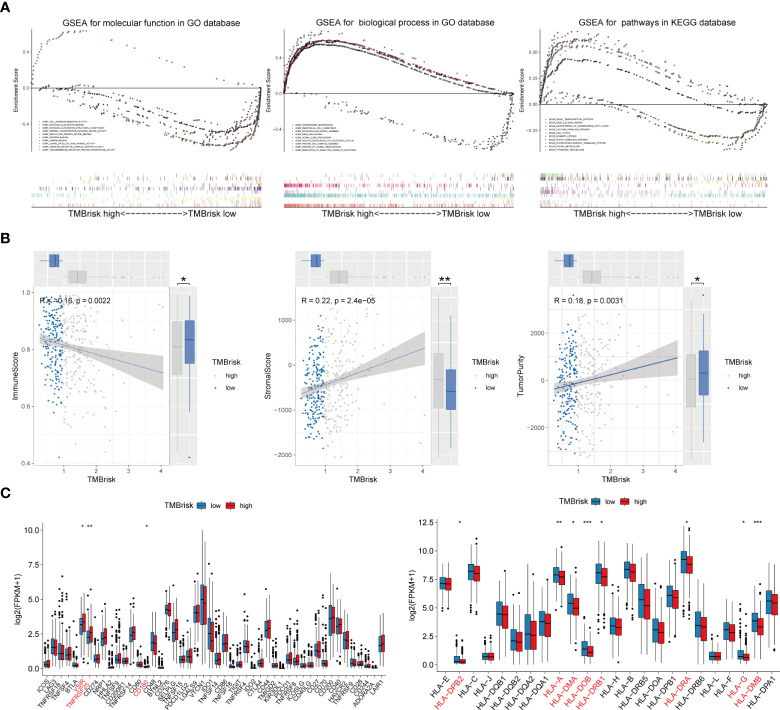
Function enrichment analysis for TMBrisk and correlation between TMBrisk and the expression of HLA family genes/immune checkpoints. **(A)** GO and KEGG enrichment of TMBrisk. In GSEA, patients were categorized into low- and high-TMBrisk groups. **(B)** Correlation between immune score, stromal score, tumor purity, and TMBrisk and their distribution in the low- and high-TMBrisk groups. **(C)** Analyses of the expression of immune checkpoints and HLA family genes in different TMBrisk groups. *p < 0.05; **p < 0.01; ***p < 0.001. TMB, tumor mutational burden; GO, gene ontology; KEGG, Kyoto Encyclopedia of Genes and Genomes; GSEA, gene set enrichment analysis.

Given that TMB has become an evolving biomarker in the field of immuno-oncology9, further exploration of the association of TMBrisk with OC immune signature is warranted. First, to determine the underlying mechanisms leading to the different outcomes between the low- and high-TMBrisk groups, we performed GSEA with annotations of GO (**Supplementary Tables 6**, **7**) and KEGG gene sets (**Supplementary Table 8**). **Figure 9A** showed that cell cycle, DNA repair, purine and pyrimidine metabolism, and biosynthesis of unsaturated fatty acids were significantly enriched in the high-TMB group, while those related to immune cell migration and immune response, such as NOTCH signaling pathway, integrin binding, laminin binding, endothelial cell chemotaxis, and phosphatidylinositol signals, were enriched in the low-TMBrisk group. In addition, immune scores were significantly lower in the high-TMBrisk group, while stromal scores and tumor purity were higher (**Figure 9B**).

**Figure 10:**
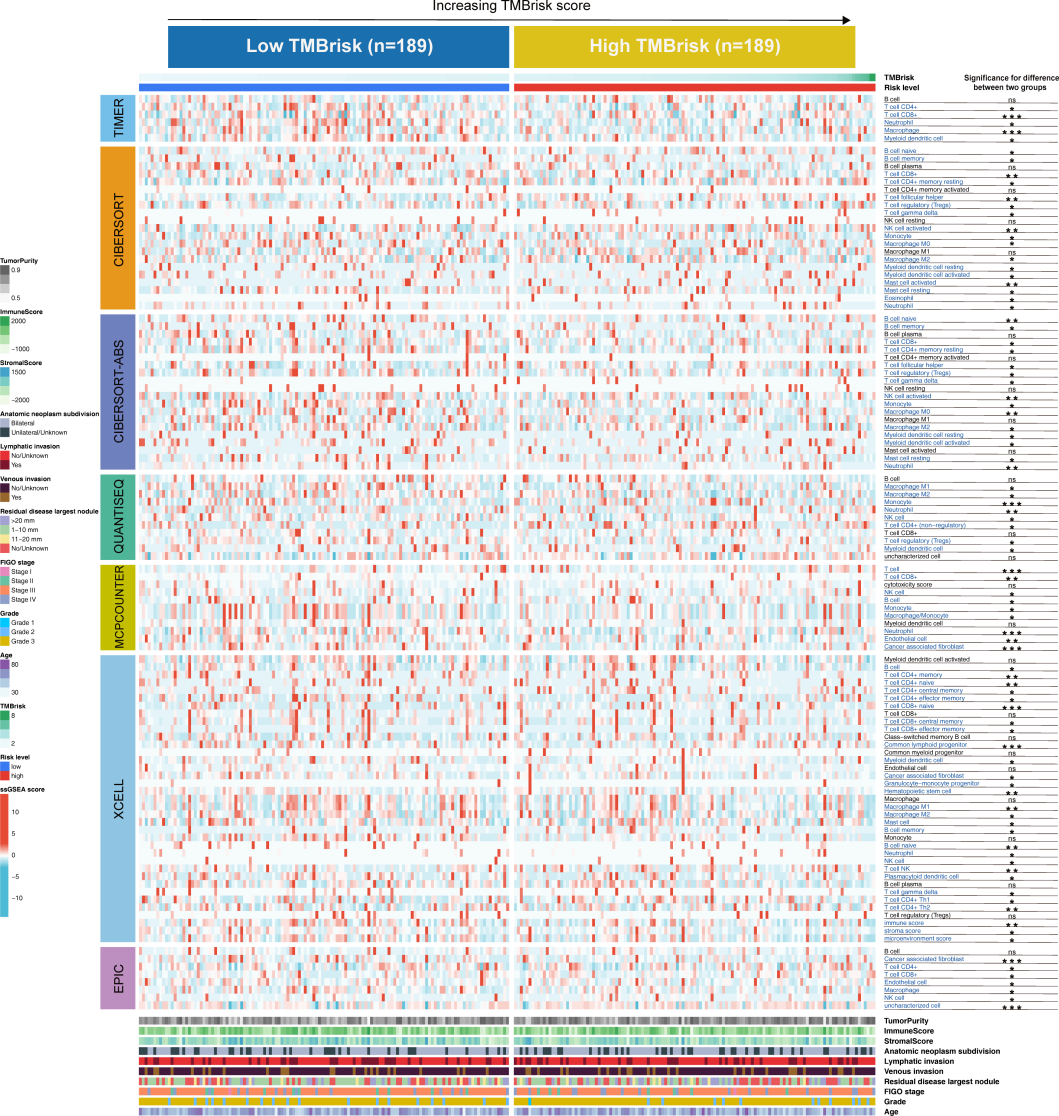
Landscape of immune and stromal cell infiltrations in the low- and high-TMBrisk groups. Heatmap showing the normalized scores of immune and stromal cell infiltrations. The statistical difference between the two groups was compared by the Wilcoxon test. *p < 0.05; **p < 0.01; ***p < 0.001. The clinical features of the patients were also described as annotations.

We also evaluated gene expression of the 48 immune checkpoints and 24 HLA family genes between the low- and high-TMBrisk groups. Our analysis showed that three immune checkpoints and eight HLA family genes were significantly modulated in the high-TMBrisk group (**Figure 9C**). The distribution of infiltrating immune cells between the low- and high-TMBrisk groups inferred by seven databases demonstrated that a majority of immunocytes decreased in the high-TMBrisk group, such as macrophages, NK cells, CD4+ T cells, and endothelial cells (**Figure 10**). However, neutrophils, and monocytes infiltrated more in the high-TMBrisk group (**Figure 10).**

### TMBrisk predicts new therapeutic regimens

To find new drugs that could be incorporated into OC treatment, we estimated the IC50 value of 138 drugs in TCGA-OV patients. We found that patients in the low-TMBrisk group tended to be more sensitive to docetaxel, mitomycin C, BMS.708163, etc., while patients in the high-TMB group tended to be more sensitive to BIBW2992, camptothecin, metformin, etc. (**Supplementary Figure 8A**). Digging deeper, the DEGs between the low- and high-TMBrisk groups were identified, and subsequently imported into CMap. In total, 20 negatively related molecular agents (enrichment < 0 and p < 0.05) for anti-OC were identified, and 21 mechanisms of action (MOA) were shared among them (**Supplementary Figure 8B**). We found that a majority of drug-target MOA belong to the inhibition class. For example, indirubin and purvalanol-a shared the mechanism of CDK inhibitor; olaparib and veliparib shared the mechanism of PARP inhibitor. In summary, our study screened drugs targeting the TMBrisk-related genes. These small molecules might potentially inhibit the occurrence of OC and need to be further studied.

## Discussion

Although immunotherapy is gaining prominence in the treatment of cancers, including OC, only a small percentage of patients respond to it. TMB is emerging as a potential biomarker of immune response and is linked with prognosis (18, 50). Its predictive performance has been verified in a variety of cancers, such as lung cancer (51), head and neck cancer (52), and melanoma (53). With regard to OC, the effect of TMB as a potential predictor and its relationship with immunotherapy responsiveness and immune infiltration still need to be further explored. In the current study, we evaluated the correlation between TMB and OC clinical traits as well as immune infiltration, screened TMB-related DEGs (including immune genes), developed a risk scoring system (TMBrisk) based on the TMB-related gene module and validated its performance externally, explored TMBrisk in respect of its immune signature, and finally predicted potential therapeutic regimens for OC.

By analyzing the somatic mutation profiles, we identified the two most frequently mutated genes in OC: TP53 (90%) and TTN (21%). As a tumor suppressor, TP53 is sufficient to regulate multiple cell cycle control networks, the loss of which will lead to dramatic gene expression changes (54). Consistent with our study, Costa et al. discovered that OC was characterized by great genomic instability with universal TP53 mutations (55). TTN, whose mutations are frequently identified in solid tumors, is associated with increased TMB, and patients with mutated TTN have improved outcomes in response to ICBs (56). We also found that SNP, which could explain a significant proportion of the heritable risk of cancer (57), is the main variant type in OC. The relationship between some types of SNPs and the occurrence, progression, and treatment of OC has been elucidated, such as the ALDH2∗2 polymorphism (rs671) (58), the most common SNP in Asia, but further study is still necessary.

Consistent with previous research of cutaneous melanoma (59), we discovered that OC patients in the high-TMB group had superior survival outcomes, and a higher TMB correlated with lower clinical stages. Interestingly, these findings were different from those in head and neck squamous cell carcinoma (52) and clear cell renal cell carcinoma (60), indicating that TMB may have distinct prognostic value and stage correlation in different tumor types. Thus, a pan-cancer analysis may be required to elucidate its role comprehensively. Furthermore, our research founded a positive correlation between TMB and immune score and a negative correlation between TMB and tumor purity, suggesting that patients with higher TMB presented more active immune activity, which may be because higher TMB results in more neo-antigens, increasing chances for T-cell recognition (61).

The relationship between TMB and immune infiltration was further explored. We demonstrated that Tregs and CD8+ T cells were downregulated, while macrophages M1 and M2 were upregulated in the high-TMB group. Tregs maintain immune homeostasis via suppressing excessive immune responses, but Tregs also infiltrate in the tumor microenvironment (TME) and inhibit antitumor immune activities (62). It is believed that Tregs are relevant to poor prognosis (63). As a result, some novel immunotherapies that deplete tumor-infiltrating Tregs can enhance antitumor effects (64, 65). These results partly explain why the high-TMB group, which possessed lower proportions of Tregs infiltration, had better OS and PFS. However, in OC, some studies have shown that higher CD8+ T-cell infiltration correlated with improved clinical outcomes (66, 67). Therefore, the mechanisms by which TMB and immune cell phenotypes influence the prognosis of OC patients may require more evidence and discussion. The M1/M2 macrophage paradigm plays a key role in tumor progression: M1 as pro-inflammation and antitumoral, and M2 as anti-inflammation, immunosuppression, and pro-tumoral (68–70). Thus, the M1/M2 ratio is critical for TME homeostasis and immune function. Studies have demonstrated that a high M1/M2 ratio status is associated with favorable prognosis for most solid tumors, including OC (69, 71, 72). In the context of increased M1 and M2 in the high-TMB group, it is necessary to further compare the M1/M2 ratio of the two groups in future studies, so as to better clarify the relationship between TMB and immune function of OC patients.

WGCNA harbors predominant advantages over other bioinformatics methods. Its clustering results (co-expression gene modules) have high biological significance and reliability. More importantly, WGCNA can quantify the correlation between co-expression modules and clinical features, so it can be used to search for gene modules of interest (73). Various studies (74–76) have applied WGCNA to cancer research and demonstrated its effectiveness. In this study, we identified the TMB-related gene module by WGCNA. The functional enrichment analysis revealed that genes in this module were mainly associated with cell cycle-related pathways. Cell cycle dysregulation is a hallmark of tumor progression, and the process of immune response is also dependent on the cell cycle (77). Recent studies have shown that cell cycle activity of both cancer and immune cells in TME could modulate immune function (78, 79).

From the green module, we screened four TMB-related immune genes significantly associated with the prognosis of OC patients. HDGF plays a vital role in the transformation, apoptosis, angiogenesis, and metastasis of cancer cells (80). Recent studies in OC showed that HDGF was passively released by necrotic and apoptotic cells; extracellular HDGF acted as a messenger of cellular condition and further enhanced cellular migration (81). In addition, higher HDGF expression was closely associated to poorer OS (82). NRAS, along with KRAS and HRAS, constitutes the RAS family, which is the most frequently mutated gene family in cancer (83). Similarly, OC has a high incidence of somatic mutations in MAPK pathway genes, the most common of which include NRAS, and MAPK inhibitors have shown efficacy for the treatment of NRAS-mutant OC (84). NR1D1 was reported to be a tumor suppressor in OC, which retarded the growth of cancer cells by abrogating the JAK/STAT3 signaling pathway (85). Other studies have shown that NR1D1 can also interact with PARP1 and BRCA1 to inhibit their associated DNA repair pathways, leading to the lethality of OC cells (86). The protein encoded by PSMD6 is a subunit of the 26S proteasome, which is co-located with DNA damage foci and involved in DNA damage response (DDR). The deficiency of PSMD6 delays DNA repair and leads to a decline in cell survival, which may offer new therapeutic approaches for cancer (87). Few studies on PSMD6 in OC suggest that PSMD6 may be a potential research target.

Nevertheless, TMB is an imperfect biomarker, partly because (1) it is costly to get bulk gene expression to calculate TMB scores, and results from different platforms require complex coordination and calibration (18), and (2) high TMB cannot be used as a biomarker in all solid cancer types and further tumor-type specific studies are warranted (88). Therefore, we constructed a prognostic model using only seven genes selected from the green module. Among the seven genes, except C3orf38, which was associated with a high TMBrisk, the remaining six (ST7L, LRFN1, LEMD1, HMGB3, CBWD1, and RFX5-AS1) genes were protective factors. These genes represented positive or negative regulation of TMB and immune activity. For instance, one study showed that C3orf38 was contained in a candidate tumor suppressor gene (TSG) locus of OC and may serve as a TSG (89). Similar to the ST7 tumor suppressor gene, ST7L can inhibit the proliferation, migration, and invasion of cancer cells (90). Yang et al. proved that the downregulated ST7L in OC activated the WNT/MAPK pathway, thereby promoting tumorigenic activity (91). As for LRFN1, a number of LRFN1-based prognostic models have been shown to be effective in a variety of cancers, including prostate cancer (92), kidney renal clear cell carcinoma (93), and OC (94). LEMD1 belongs to the CTA family, which is a useful target of immunotherapy (95). Guo et al. have demonstrated that LEMD1 antisense RNA 1 (LEMD1-AS1) can suppress OC progression and can be used as a biomarker to predict survival (96). HMGB proteins are closely related to DNA damage repair, among which HMGB3 is often overexpressed up to 20 times in cancer cells (97). Mukherjee et al. indicated that targeted elimination of HMGB3 reduced cisplatin resistance in OC cells, increasing tumor cell sensitivity to chemotherapy (98). However, the remaining hub genes, CBWD1 and RFX5-AS1, are poorly investigated in OC, which could be topics for further study.

The advantage of this study is that it fully evaluates the robustness of the TMBrisk model, as it has been validated on three independent datasets. ROC curves and DCA showed that TMBrisk had good accuracy in predicting OS, better than other independent predictors. Currently, FIGO staging is the most widely used system for predicting the malignancy and progression of OC. However, the weakness of this system is that it mainly considers distant metastasis, lymph node invasion, tumor size and location, ignoring the heterogeneity of other clinical features (3, 99). In this study, based on TMBrisk, by incorporating other independent prognostic factors, we developed a nomogram with higher net benefit and better predictive accuracy, which can complement the FIGO system to exert a more profound clinical value.

Regarding the relationship between TMBrisk and immune status of OC patients, our study found that high TMBrisk tended to predict an immune-suppressed status. Lower immune activities were revealed in the high-TMBrisk group, including reduced immune cell infiltration and downregulation of some HLA family genes. For example, NK cells and CD4+ T cells showed low infiltration in the high-TMB group. NK cells have unique properties, in that they can swiftly kill tumor cells and enhance antibody as well as T-cell response, which support its role as anticancer agents (100). In OC, boosting NK cell expansion and functionality has emerged as an attractive therapeutic approach and has been extensively investigated (101, 102). CD4+ T cells play a critical role in the immune system and make great contribution to the antitumor response (103). It is reported that OC patients with dense infiltration of CD4+ T cells experience favorable OS and PFS (104). In addition, three immune checkpoint genes (VSIR, TNFRSF25, and CD160) were upregulated in the high TMB group, suggesting that these immune checkpoints may be potential therapeutic targets for OC similar to PD-1/PD-L1.

Finally, we identified some untraditional antitumor compounds, such as BMS.708163, metformin, and BIBW2992, with potential advantages for patients who might not gain improvement from conventional drugs. The efficacy of these drugs for OC is expected for further investigation.

Although our model proved to be valuable in determining prognosis and guiding treatment in OC patients, it should be prospectively confirmed by large-sample clinical studies. This study is a combination of bioinformatics analysis and a small number of in vitro experiments. Consequently, more experiments are warranted to explore and verify the relationship and molecular mechanisms between TMB/TMBrisk and immune infiltration. In addition, restrained by the lack of data of OC patients undergoing immunotherapy, unfortunately, it was not possible to assess whether patients with different TMBrisk benefited differently from immunotherapy. In our further work, studies should be conducted to compare TMBrisk with current biomarkers and explore the relationship between TMBrisk and immunotherapy in OC patients.

## Data availability statement

The datasets presented in this study can be found in online repositories. The names of the repository/repositories and accession number(s) can be found below: https://figshare.com/, https://doi.org/10.6084/m9.figshare.19729651.v2. 

## Author contributions

Research design: HW, JL, and LH. Data collection: JP and YW. Data analysis: HW, JL, and JY. Manuscript preparation: HW and JL. Chart preparation: HW, ZW, and ZZ. Revisions: HW, JL, JY, and LH. All authors confirm that they contributed to manuscript reviews, critical revision for important intellectual content, and read and approved the final draft for submission. All authors are also responsible for the manuscript content.

## Conflict of interest

The authors declare that the research was conducted in the absence of any commercial or financial relationships that could be construed as a potential conflict of interest.

## Publisher’s note

All claims expressed in this article are solely those of the authors and do not necessarily represent those of their affiliated organizations, or those of the publisher, the editors and the reviewers. Any product that may be evaluated in this article, or claim that may be made by its manufacturer, is not guaranteed or endorsed by the publisher.
