# Supplementary figures and images for: A novel tumor mutational burden-based risk model predicts prognosis and correlates with immune infiltration in ovarian cancer
Source: Front Immunol. 2022 Aug 8;13:943389. doi: 10.3389/fimmu.2022.943389 (PMC9393426; doi:10.3389/fimmu.2022.943389)

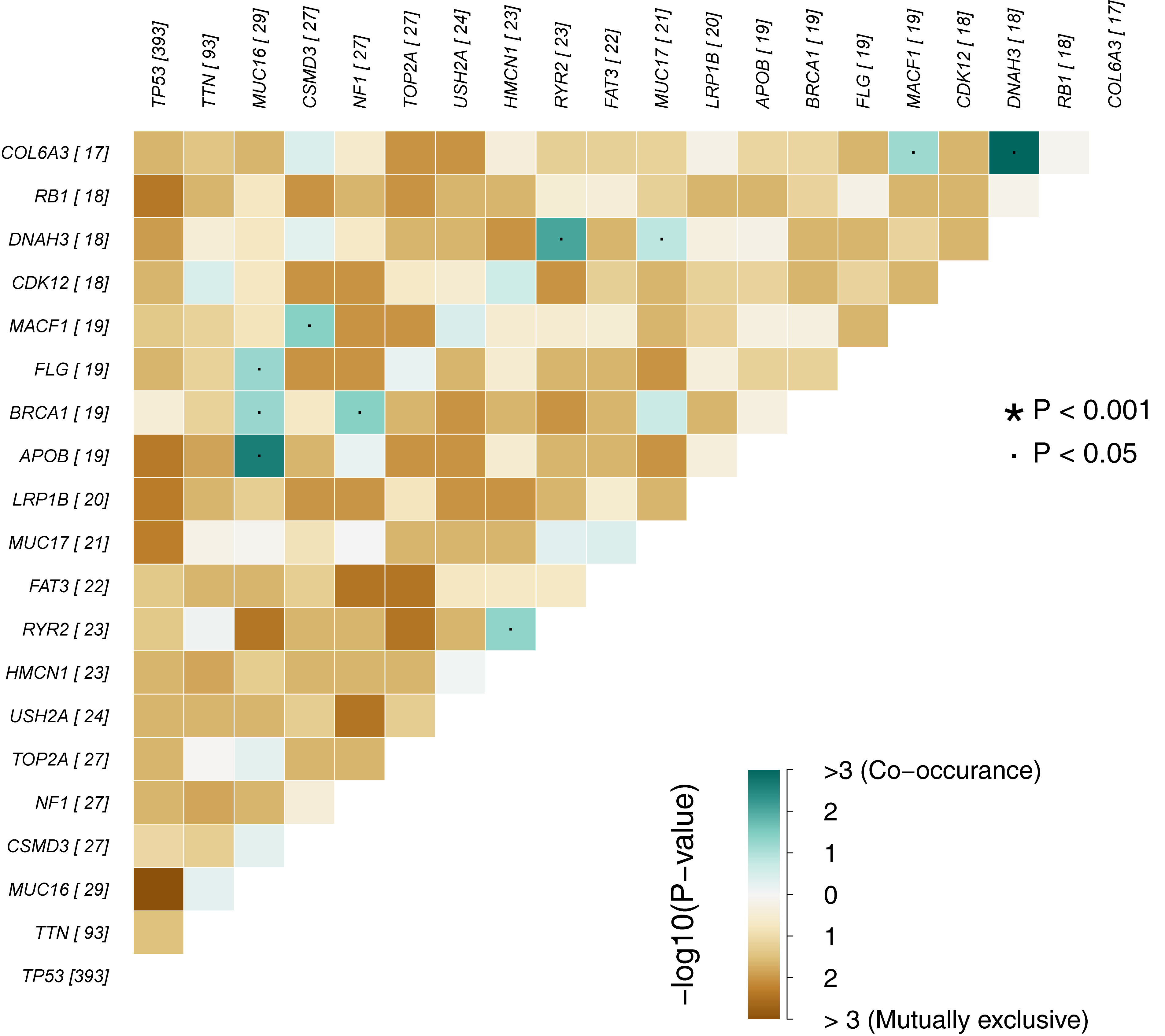

Supplement: Supplementary file 1 [file Image_1.tif]

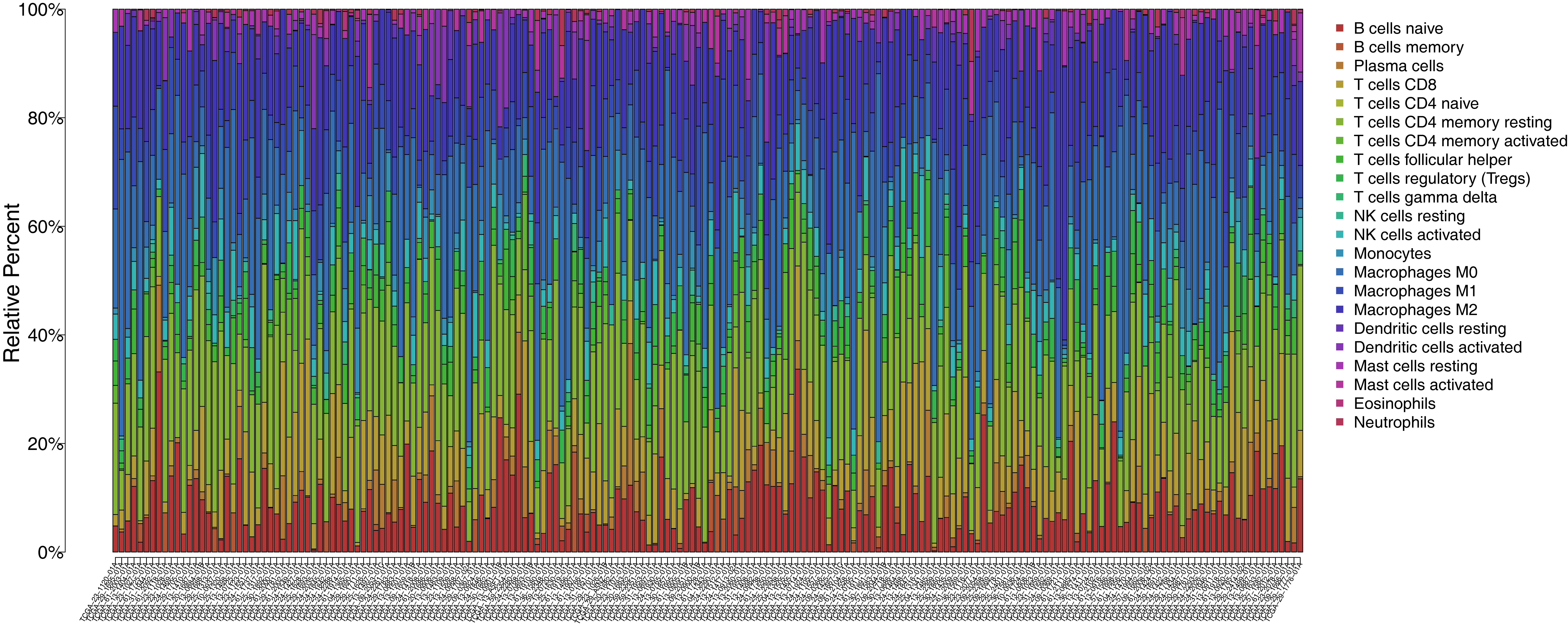

Supplement: Supplementary file 2 [file Image_2.tif]

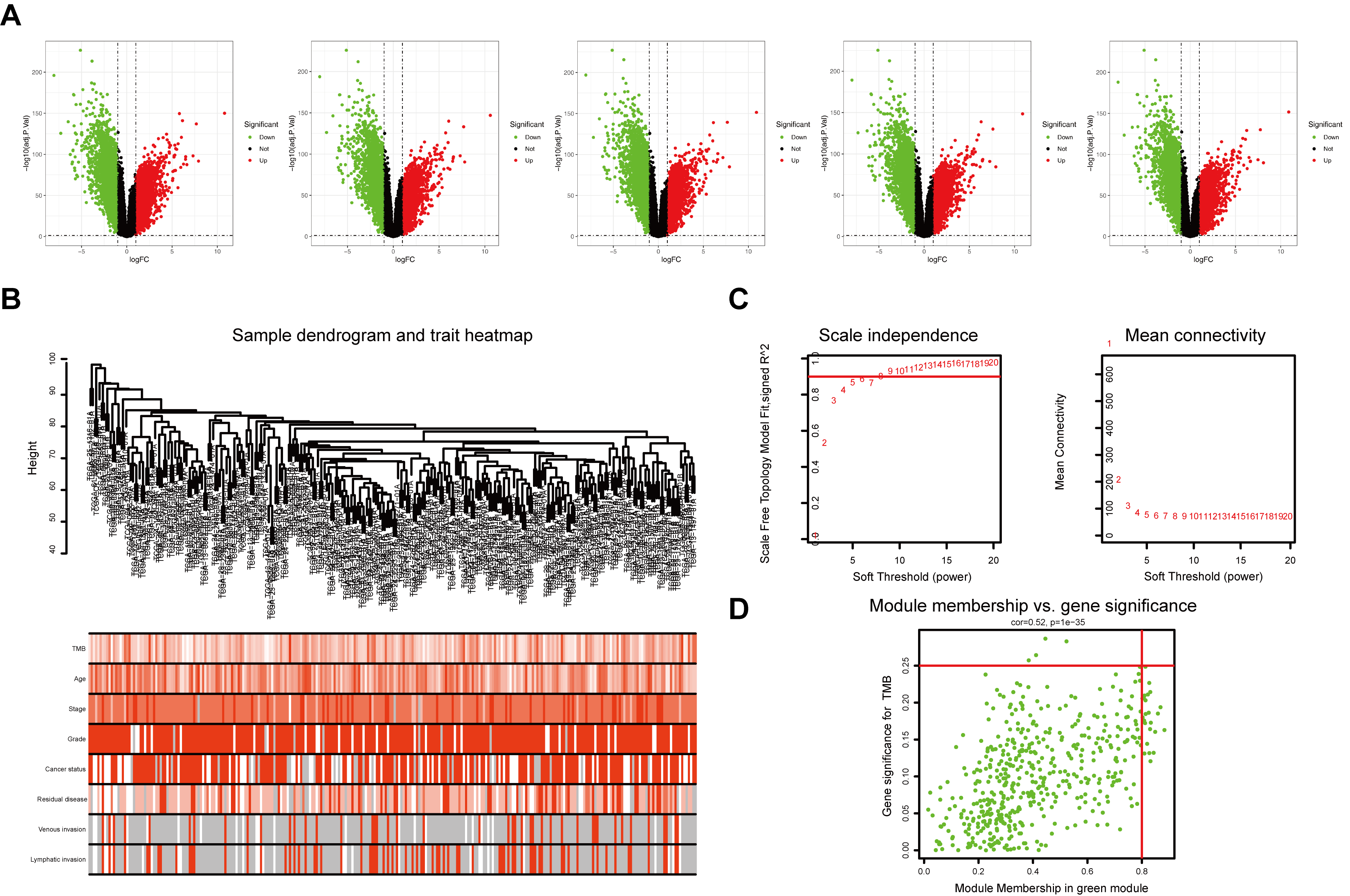

Supplement: Supplementary file 3 [file Image_3.tif]

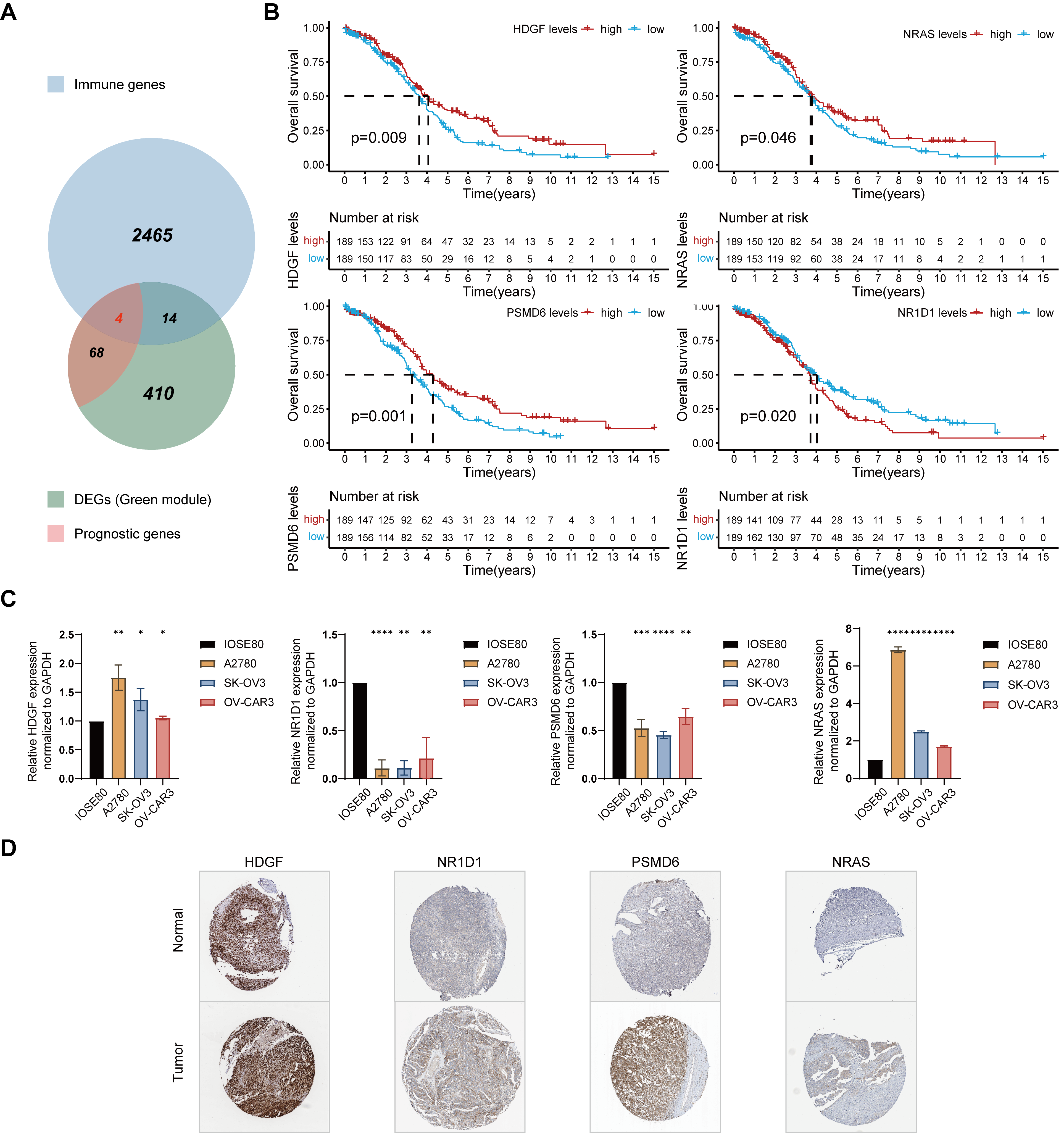

Supplement: Supplementary file 4 [file Image_4.tif]

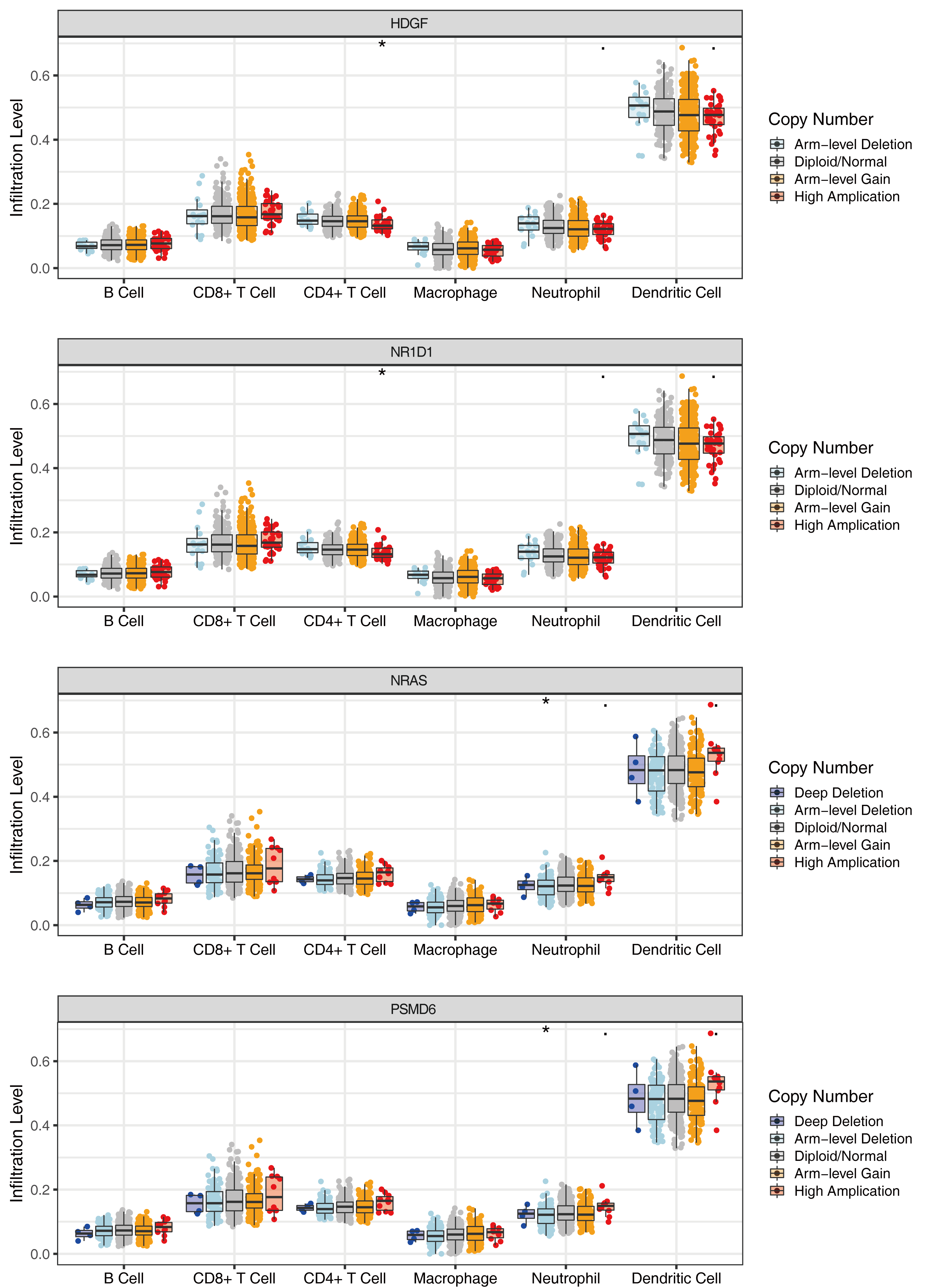

Supplement: Supplementary file 5 [file Image_5.tif]

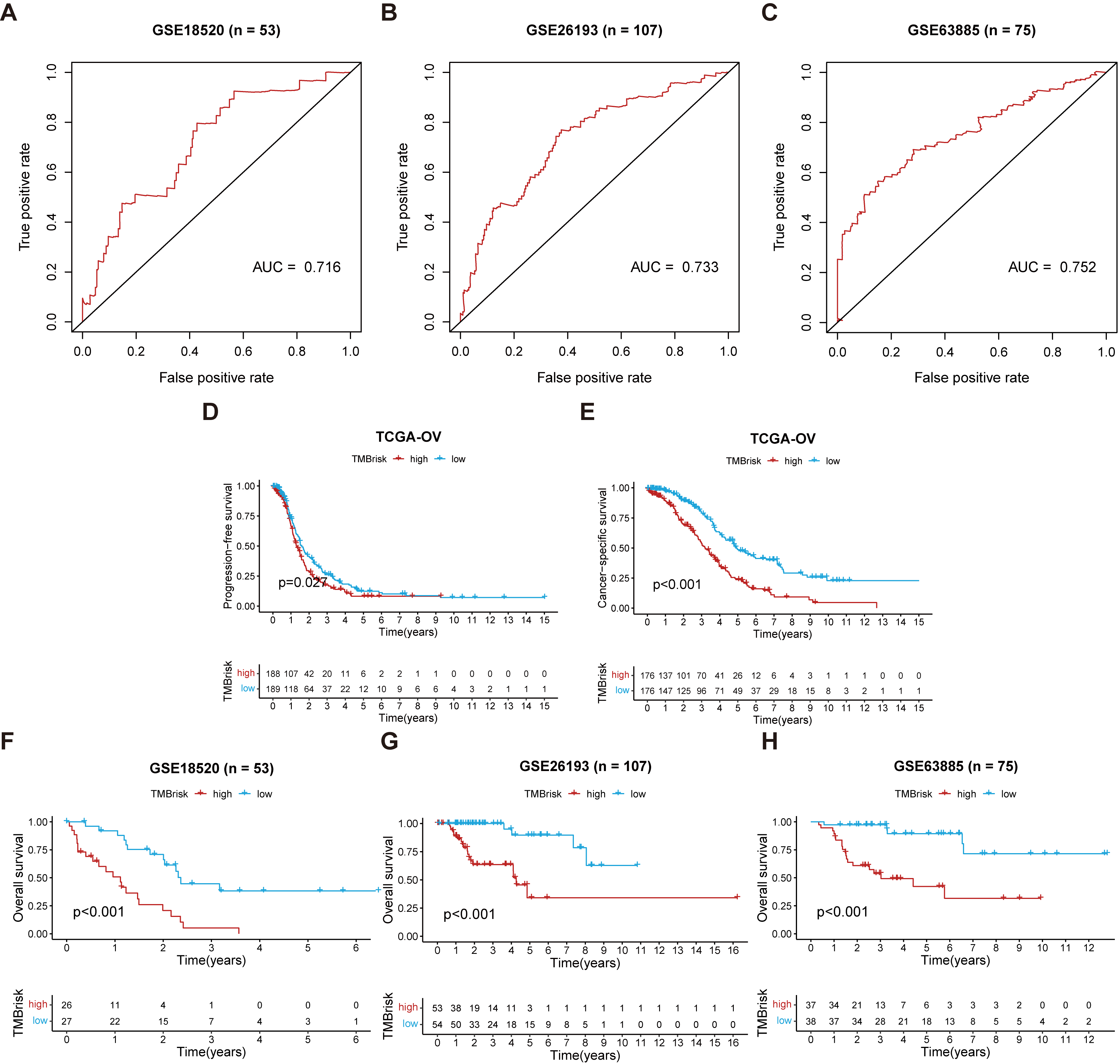

Supplement: Supplementary file 6 [file Image_6.tif]

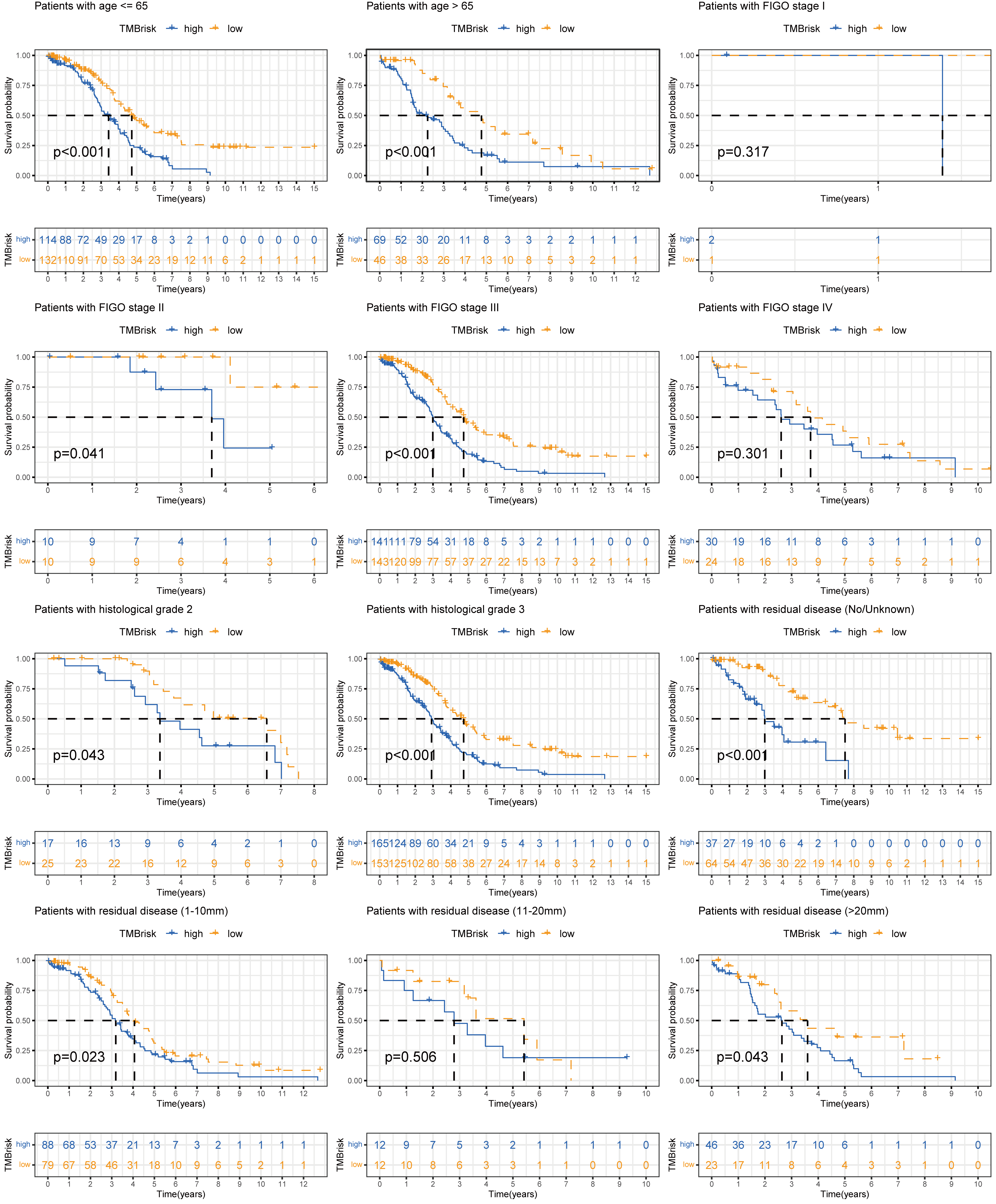

Supplement: Supplementary file 7 [file Image_7.tif]

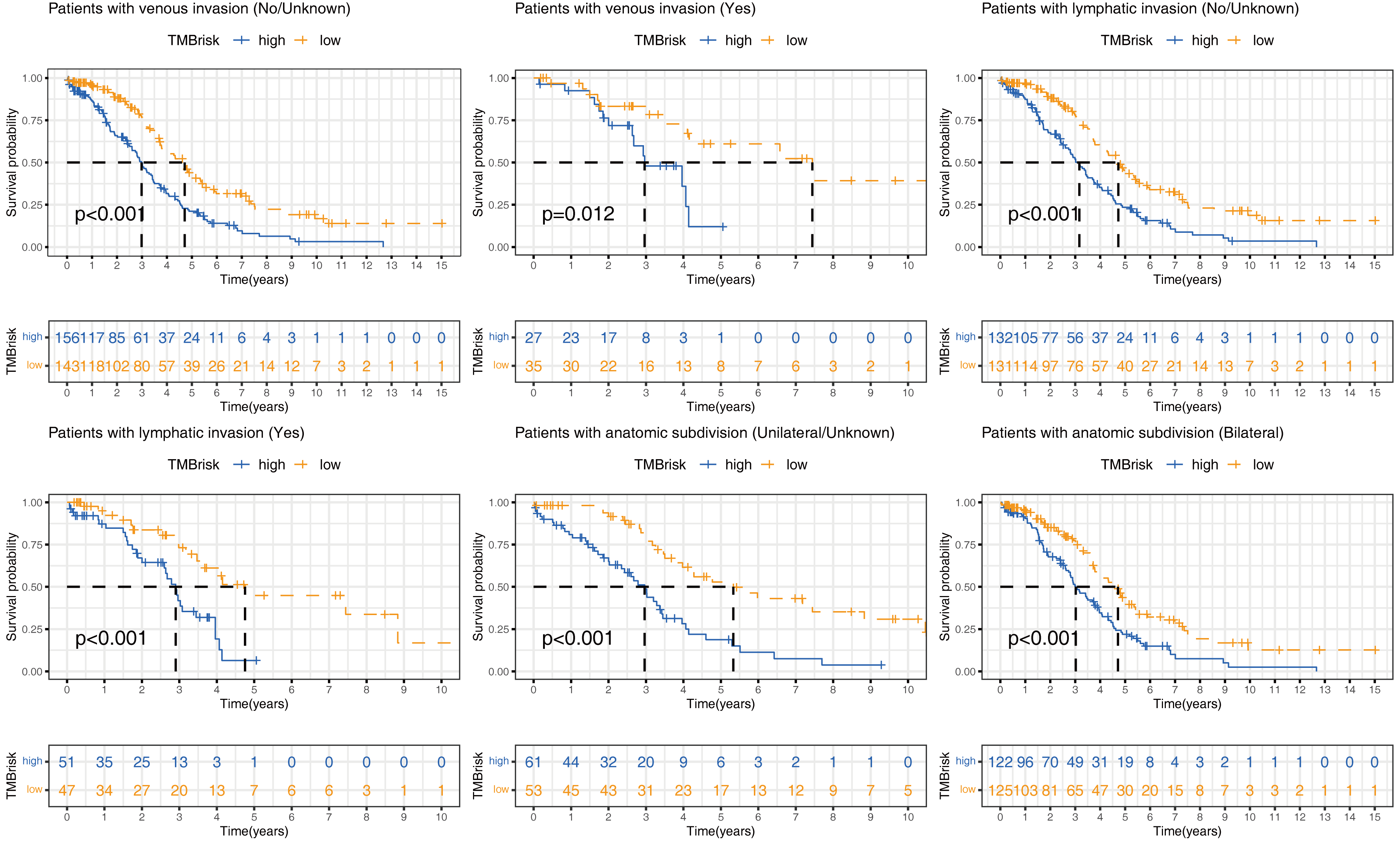

Supplement: Supplementary file 8 [file Image_8.tif]

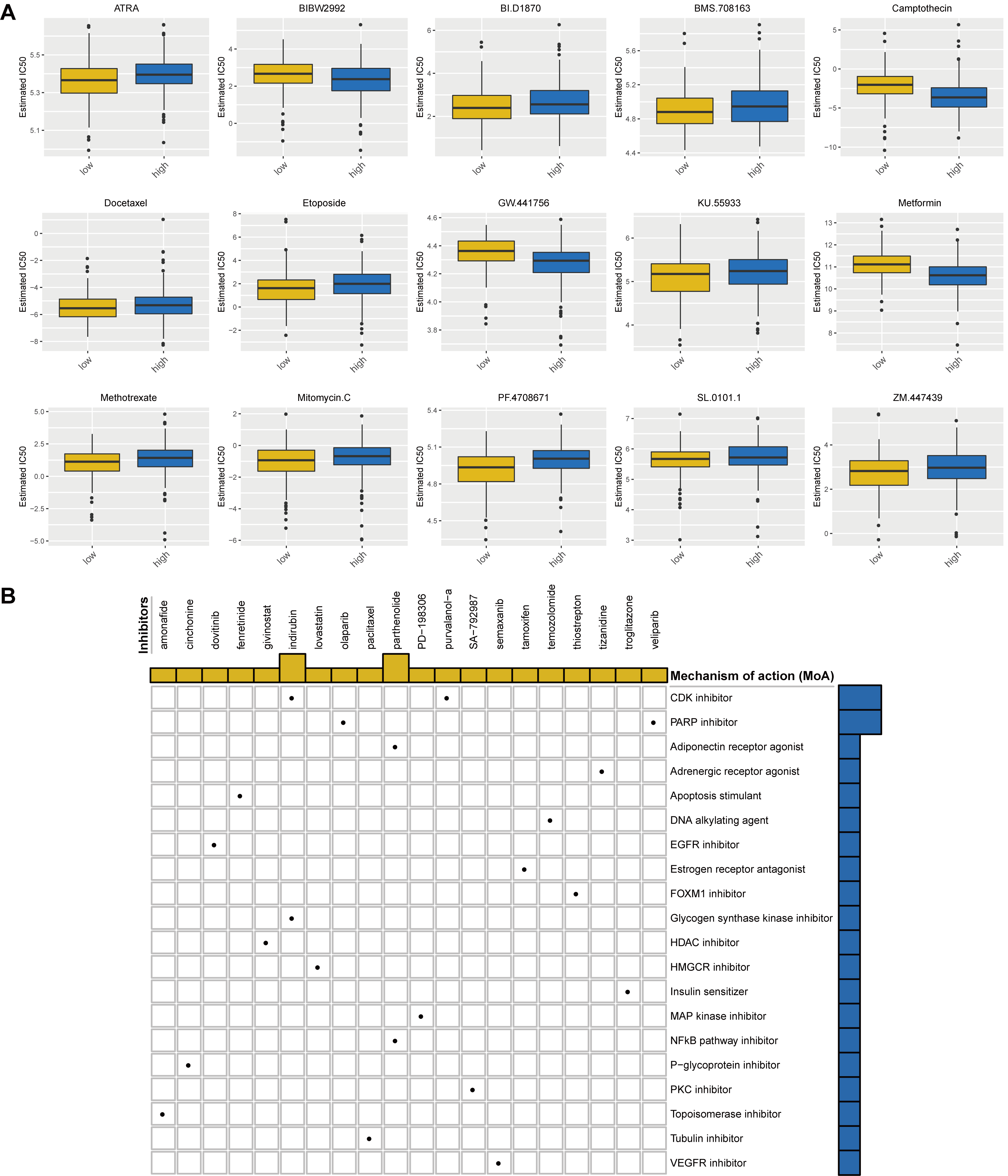

Supplement: Supplementary file 9 [file Image_9.tif]
