# Supplementary material for: A novel tumor mutational burden-based risk model predicts prognosis and correlates with immune infiltration in ovarian cancer
Source: Front Immunol. 2022 Aug 8;13:943389. doi: 10.3389/fimmu.2022.943389 (PMC9393426; doi:10.3389/fimmu.2022.943389)
Supplement: Supplementary file 10 [file Table_1.docx]

Supplementary Table 1. The primers of genes measured using RT-qPCR.

| **Gene** | **Forward primer (5’ → 3’)** | **Reverse primer(5’ → 3’)** |
| --- | --- | --- |
| HDGF | GAGGAGGAGGATGAAGAGGAAGAGG | TGAGTAGAAGAGGAGAGCAGGTTGG |
| NR1D1 | CCCGACCCTCCTTGCTACCTTC | TGATGACGCCACCTGTGTTGTTG |
| PSMD6 | GGAGGAGACTGGGACAGGAGAAAC | GATACTGCCGAACTGCTGGAAGAC |
| NRAS | AGCCAAGACCAGACAGGGTG | GTCAGGACCAGGGTGTCAGT |
| CBWD1 | TCCCATTGAGACGACGCAAAGC | TTCACTGAACAGCAGAGGCAACC |
| ST7L | GGGCTAGGTTAAGGCTGGCATTC | GCAGTCAGGGCTCCATTTAGTTCC |
| RFX5-AS1 | AGCTGCCGGAAGAGAAGAAT | GCGAGTAAGTGTGTCTGCAG |
| C3orf38 | GCCAGAGCCAGTTACAAAGACAGAG | GTCCCAGCAACTCCAACCATAACC |
| LRFN1 | GAACCTAAACACCCTCACGC | GGTTGGAGGTCATGTCCAGA |
| LEMD1 | ATGGTGGATGTGAAGTGTCTGAGTG | TGATTCTGGTGCTTGGTGCTCTTG |
| HMGB3 | CACCGTCTGGATTCTTCCTGTTCTG | TCCTCCTCCTCCTCCTCCTCTTC |
| β-actin | CTCGCCTTTGCCGATCC | TCTCCATGTCGTCCCAGTTG |
